# Another tail of two sites: activation of the Notch ligand Delta by Mindbomb1

**DOI:** 10.1186/s12915-025-02162-6

**Published:** 2025-03-06

**Authors:** Nicole Vüllings, Alina Airich, Ekaterina Seib, Tobias Troost, Thomas Klein

**Affiliations:** Institute of Genetics, Heinrich-Heine-Universitaet Duesseldorf, Universitaetsstr. 1, Duesseldorf, 40225 Germany

**Keywords:** Notch-pathway, Delta, DSL-ligands, Endocytosis, Mindbomb1, Ubiquitylation, Cis-inhibition

## Abstract

**Background:**

Notch signalling plays a crucial role in many developmental, homoeostatic and pathological processes in metazoans. The pathway is activated by binding of the ligand to the Notch receptor, which changes the conformation of the receptor by exerting a pulling force. The pulling force is generated by the endocytosis of the interacting ligand into the signal-sending cell. Endocytosis of ligands requires the action of the E3 ligases Mindbomb1 (Mib1) and Neuralized (Neur) that ubiquitylate lysines (Ks) of their intracellular domains. It has been shown that human MIB1 binds JAGGED1 (JAG1) via a bipartite binding motif in its ICD. This interaction is required for the activation of JAG1. However, it is not known whether this bipartite binding mode is of general importance. It is also not rigorously tested whether it occurs in vivo. Moreover, it is not known whether Mib1 ubiquitylates specific Ks in the ICD of ligands, or is rather non-selective.

**Results:**

We therefore investigated how Mib1 interacts with the Notch ligand Delta of *Drosophila* in an in vivo trans-activation assay and determined the Ks which are required for signalling. We show that the activation of Dl by Mib1 follows similar rules as has been found for mammalian MIB1 and JAG1. We present evidence that a combination of six Ks of the ICD is required for the full signalling activity of Dl by Mib1, with K742 being the most important one.

**Conclusions:**

Altogether, our analysis further reveals the rules of Mib1-mediated DSL-ligand-dependent Notch-signalling.

**Supplementary Information:**

The online version contains supplementary material available at 10.1186/s12915-025-02162-6.

## Background

Juxtacrine Notch signalling plays a critical role in many developmental, homoeostatic and pathological processes in metazoans [1]. It is initiated by transmembrane proteins of the DSL protein family as ligands and requires endocytosis of these ligands bound to the Notch receptor in trans (reviewed in [2]). This activating endocytic event depends on the ubiquitin binding endocytic adapter Epsin and creates a pulling force that forces a conformational change in Notch. The conformational change exposes a cleavage site for the metalloprotease ADAM10, encoded by *kuzbanian* (*kuz*) in *Drosophila*. The S2-cleavage by Kuz sheds the extracellular domain, which is endocytosed with the bound ligand into the signal-sending cell (reviewed in [3]). The result of S2-cleavage induced ecto-domain shedding is a membrane inserted truncated Notch molecule, termed Notch EXtracellular Truncation (NEXT), which undergoes rapid intramembranous cleavage by y-secretase (S3-cleavage). The released intracellular domain (NICD) travels into the nucleus, associates with the CSL transcription factor Suppressor of Hairless (Su(H)) to initiate the expression of the target genes of the pathway.

One major way to initiate endocytosis of the ligands is the ubiquitylation (ubi) of lysines (Ks) in their intracellular domains (ICDs) by E3 ligases (reviewed in [2, 4]). Two E3-ligases have been identified that ubiquitylate the *Drosophila* DSL-ligands Serrate (Ser) and Delta (Dl) during Notch signalling, termed Mindbomb1 (Mib1) and Neuralized (Neur) [5]. Both are necessary in complementary Notch-dependent processes for the full signalling activity of the ligands. Dl directly binds Mib1 and Neur at different sites of its ICD and is ubiquitylated by these two E3s on Ks [6, 7]. For the mammalian Ser ortholog JAGGED1 (JAG1), the binding mode of MIB1 has been determined at the atomic level. It binds to two short epitopes in the ICD of JAG1, termed N- and C-box (NB and CB, respectively) with its N-terminal MZM and REP domains, respectively [7]. This bipartite binding mode is required for the full function of JAG1 in cell culture assays and in *Drosophila *in vivo experiments [7].

**Fig. 1:**
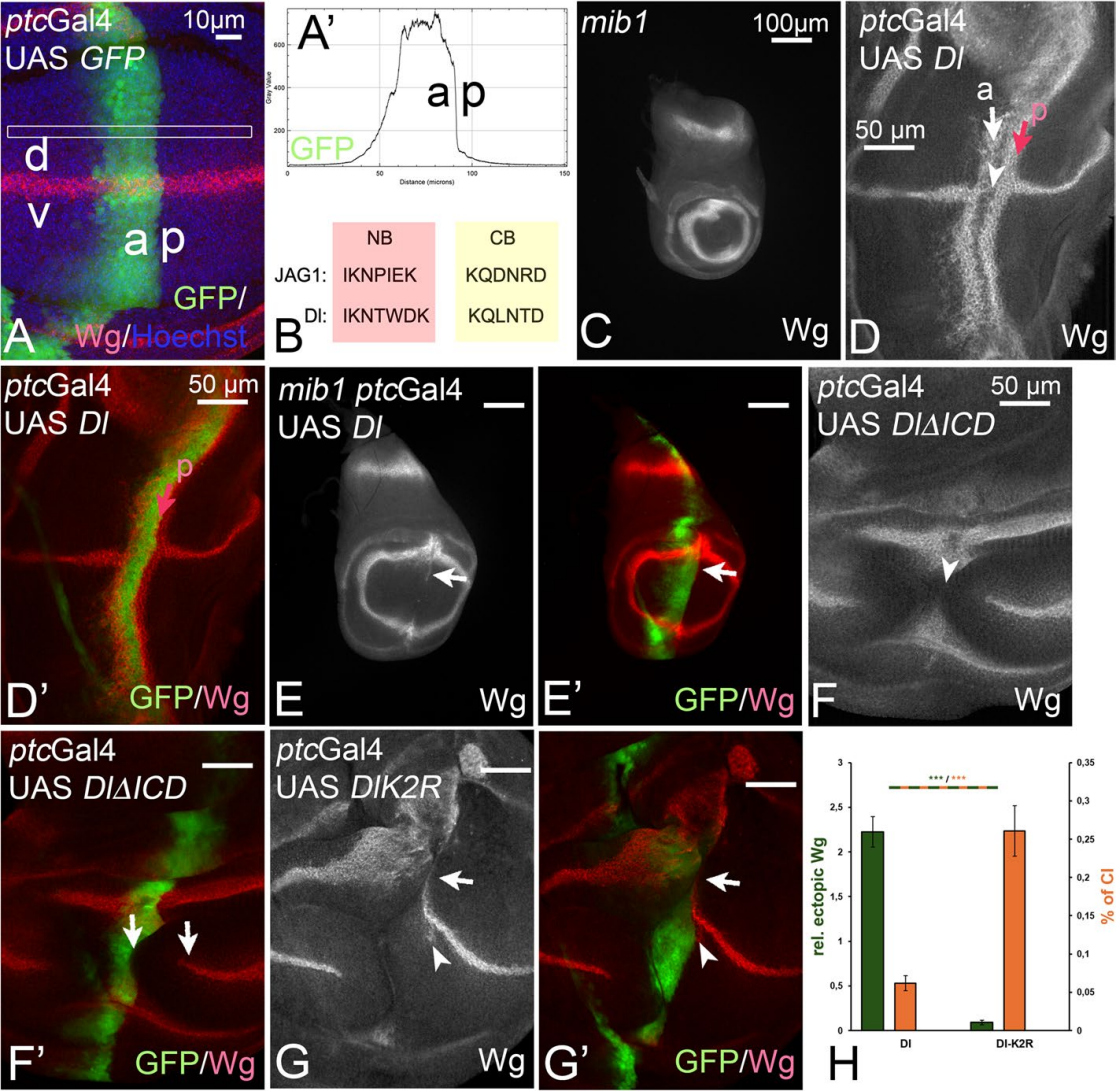
Effects of expression of DlΔICD and DlK2R during wing development. **A** The constructs are expressed with ptcGal4, which drives expression in a stripe along the anterior side (a) of the A/P-boundary of the wing imaginal disc. Expression in the wing disc is perpendicular to the D/V-boundary (d, v) along which the Notch target gene wg is expressed. **A’** Expression is graded within the ptcGal4 domain, increasing from anterior to the A/P-boundary. The gradient is measured by the pixel intensity of the GFP-signal in the region boxed in **A**. **B** The sequence comparison of the NB and CB of JAG1 with Dl. **C** Expression of Wg in mib1 mutant discs. The expression along the D/V-boundary is lost and the wing area is dramatically reduced. **D**, **D’** Ectopic expression of Dl with ptcGal4 in wildtype discs results in the induction of two stripes of ectopic Wg expression running perpendicular to the endogenous expression along the D/V-boundary. One anterior broader stripe (arrow, a) and a thinner stripe located in the posterior boundary cells (red arrow, p). At the intersection with the D/V-boundary, the endogenous expression of Wg is cell-autonomously suppressed in the domain of high expression due to CI (arrowhead). The cell-autonomy of CI is revealed by the expression of wg directly adjacent to the posterior boundary of the ptcGal4 domain (red arrow). **E**, **E’** Expression of Dl in a mib1-mutant wing disc results in a very weak activation of Wg in the dorsal compartment of the wing area (arrow), confirming that Dl can signal weakly in the absence of Mib1 function, but requires Mib1 function for its full activity (compare with **C**, **D**, **D’**). **F**, **F’** Expression of DlΔICD causes a large gap in the endogenous expression of Wg (arrowhead in **F**). A gap can be observed between the posterior expression boundary of the ptcGal4 domain and the endogenous expression of Wg (arrows in **F’**). This non-cell autonomous suppression of Wg expression indicates that DlΔICD acts in a dominant-negative manner. No ectopic expression of Wg is induced, indicating the activity of Dl is abolished if the ICD is deleted. **G**, **G’** Expression of DlK2R results in a suppression of the endogenous expression of Wg. Note that this effect is restricted to the domain of expression and accompanied by weak ectopic activation of Wg in posterior boundary cells in the dorsal compartment (arrow and arrowhead). This indicates that DlK2R is weakly active and possess stronger cis-inhibitory abilities than Dl, indicated by the larger gap in the endogenous expression domain of Wg (arrow and arrowhead) [8]. **H** Quantification of the signalling activity and CI of Dl and DlK2R (see Fig. 2 and Methods for more details). It confirms that the loss of the Ks results in a dramatic reduction in signalling, accompanied by a strong increase in CI

Corresponding boxes, termed ICD2 (= NB) and ICD3 (= CB), have been identified in the ICD of Dl and it has been shown that also the NB/ICD2 of Dl binds to the MZM domain of MIB1 in vitro and is important for the in vivo function of Dl [6, 7] (Fig. 1G). The NB/ICD2 appears to constitute the major binding site of Dl, since a Dl-variant that lacks the NB could not be co-immunoprecipitated with Mib1 [6]. It appears that, in contrast to JAG1, the CB (ICD3) is not required for Mib1-dependent signalling of Dl, although it is conserved among insect species [6]. Thus, it is not clear whether the bipartite binding mode determined for the interaction between MIB1 and JAG1 applies to the interaction of Mib1 with other ligands.

In the wing imaginal disc of *Drosophila*, Mib1 is expressed ubiquitously, while Neur is restricted to single, late arising single sensory organ precursor cells (SOPs) of the peripheral nervous system. Thus, by far the most DSL-signalling (e. g. during wing development) is mediated by Mib1. The loss of function of *mib1* in the wing disc results in a strong reduction of Notch activity, which causes the loss of most parts of the wing anlage and the loss of expression of wing-specific target genes, such as *wingless* (*wg*) along the dorso-ventral (D/V)-compartment boundary [9–11].

Besides the productive interaction of Notch and Dl in trans, both proteins are also engaged in cis-interactions in the same cell, which cause the cell-autonomous suppression of Notch signalling. This phenomenon is termed cis-inhibition (CI) and is exploited in several developmental processes to regulate the signalling activity of the pathway and enables directional signalling [12–14]. CI inversely correlates with the efficiency of a ligand to be endocytosed [8, 15, 16].

We have previously tested the requirement of ubi for the function of Dl by replacing all its Ks of its ICD by the structurally similar arginine (R, DlK2R) [8, 16]. DlK2R cannot be ubiquitylated by Mib1 and Neur [6, 8]. By analysing the activity of DlK2R, we found that Dl-signalling can be activated in three different ways: one completely independently of ubi and both E3-ligases, one dependent on Neur, but independent of ubi and one dependent on Neur- and Mib1-mediated ubi [8, 16]. During signalling events, the three ways add up to the full activity of Dl. By analysing a DlK2R knock-in allele (*Dl*^*attP*^-*DlK2R*), we recently found that the two ubi-independent modes combined allow the complete development of *Drosophila* [16]. However, the adult *Dl*^*attP*^-*DlK2R* flies displayed Notch-related defects, indicating the requirement of Mib1-mediated ubi for the full function of Dl. The previous work also indicates that, in contrast to Neur, Mib1 activates Dl solely by ubi of its ICD on Ks [8, 16].

In general, E3-ligases ubiquitylate the Ks of its substrates either non-selectively or selectively on distinct Ks [17]. In the case of mouse Delta-like1 (Dll1), a single K, K613, has been identified as the major site of ubi in cell culture experiments, suggesting that MIB1 is highly selective [18]. However, previous attempts to identify individual Ks in the ICD of *Drosophila* Dl relevant for its activation by Mib1 failed [6]. Moreover, the deletions of individual conserved Ks did not have a measurable impact on ubi of the ligand by Neur and Mib1 [6].

Here, we present the results of our analysis that further investigated the activation of Dl by Mib1. We show that, besides the NB, also the CB predicted in Dl is involved in signalling in vivo. We provide in vivo evidence that the MZM and REP domains of *Drosophila* Mib1 bind to the NB and CB of Dl, respectively. Thus, the activation of Dl by Mib1 follows similar rules as determined for the interaction between human MIB1 and JAG1. Moreover, we show that a combination of six Ks in the ICD is required for the full activation of Dl by Mib1, with K742 being the most important one. Altogether, our analysis reveals the rules of Mib1-mediated DSL/Notch signalling.

## Results

For the initial analysis, we used the Gal4 expression system to ectopically express our generated Dl-variants. All constructs were inserted in the same landing site to neutralise position effects on their expression and guarantee comparable expression. *patched*-Gal4 (*ptc*Gal4) induces expression of UAS constructs in a band of cells along the anterior side of the anterior–posterior compartment boundary (A/P-boundary) of the wing imaginal disc (Fig. 1A). The expression within the domain increases in a sigmoidal curve towards the posterior expression boundary, which is identical to the A/P-boundary (Fig. 1A’). Note that only Mib1 is present during wing development with the exception of single late arising expressing neural precursor cells that express Neur. Thus, the assay in the wing disc specifically addresses Mib1-mediated activation of Dl.

We used the expression of Wg as a read-out for Notch activity. It is expressed in a stripe that straddles the dorso-ventral compartment boundary (D/V-boundary) as a result of continuous Notch signalling (reviewed in [9]) (Fig. 1A). The loss of *mib1* function results in the loss of Wg expression along the D/V-boundary (Fig. 1C). Ectopic expression of Dl with *ptc*Gal4 in wildtype discs results in the induction of ectopic expression of the target gene *wg* in two stripes perpendicular to its normal domain along the D/V-boundary [12, 19] (Fig. 1D, D’). As previously reported, its expression with *ptc*Gal4 interrupted the endogenous expression of Wg along the D/V-boundary not only at the point of intersection with the *ptc*Gal4 domain, but also in adjacent non-expressing posterior cells [20] (Fig. 1D, D’, arrowhead and arrows, respectively). Expression of Dl in *mib1* mutant discs induced only a very weak short stripe of Wg in a non-penetrant manner, indicating the requirement of Mib1 for signalling of Dl in wing discs (Fig. 1E, E’, arrow). We first confirmed the meaning of the ICD of Dl by generating a variant, DlΔICD-HA, which lacks most of the ICD, but is still inserted in the plasma membrane. This non-cell autonomous behaviour indicates that DlΔICD-HA is a dominant-negatively acting variant. The dominant-negative behaviour is different from the cis-inhibitory suppression of the expression of Wg by active Dl-variants, e.g. Dl and also DlK2R, which is cell-autonomous, as it is restricted to the *ptc*Gal4 expression domain [19, 21, 22] (Fig. 1F, F’, compare with 1D–E’). The different phenotype produced by DlΔICD-HA compared to Dl and DlK2R-HA highlights the importance of the ICD for the function of Dl and also confirms that Dl can weakly signal in the absence of Ks in its ICD [8]. This ubi-independent signalling explains the finding that expression of Dl in *mib1* mutants slightly induces the expression of Wg in a non-penetrant manner (Fig. 1E, E’, arrow) [8]. To quantify the effects of over-expression of the Dl-variants, we measured the length of the anterior and/or posterior ectopic Wg expression induced and divided it by the length endogenous wg expression along the D/V-boundary to account for the variation in disc size. Ten discs were measured for each genotype (Fig. 1H and subsequent figures). For the strength of CI, we measured the length of the gap within the endogenous expression of Wg along the D/V boundary and related it to the total length of the Wg domain. The quantification of the signalling properties and cis-inhibition confirmed that the signalling abilities of DlK2R were dramatically reduced, while its CI was strongly increased. This inverse correlation was observed previously and indicates that the Ks are important to adjust CI to the correct level [8, 16].

### Dl has a functional CB required for Mib1-dependent activation

**Fig. 2:**
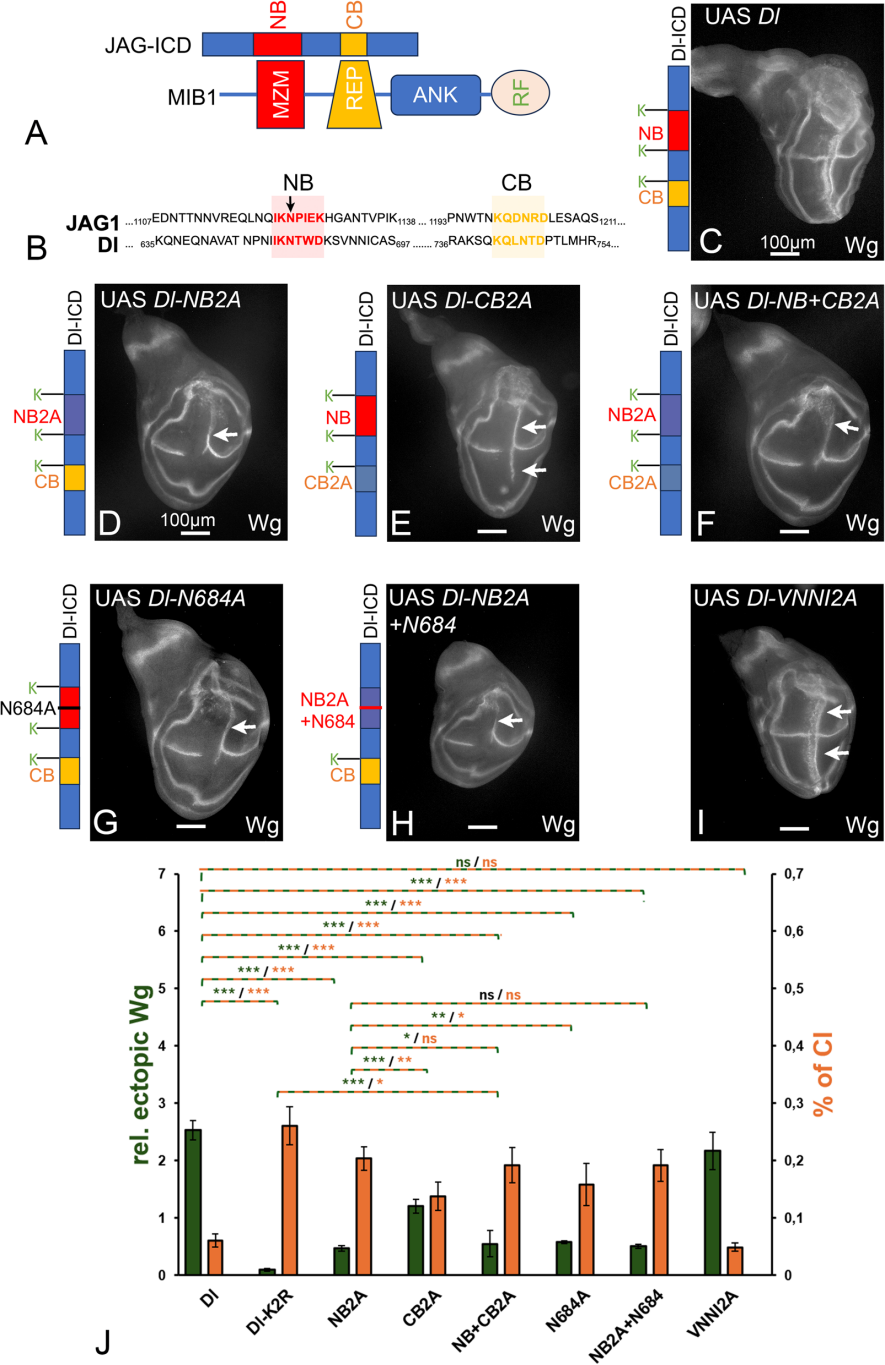
The importance of the NB and CB for the activity of Dl. **A** Cartoon of the bipartite interaction mode between JAG1 and MIB1. The NB and CB of JAG1 interact with the MZM and REP domain of MIB1, respectively. **B** Sequence comparison of sections of the ICDs of JAG1 and Dl that include the NB/ICD2 (shaded in red) and the predicted CB/ICD3 (shaded in yellow). The arrow points to the conserved asparagine (N) in the NB. **C** Expression of Dl by ptcGal4 for comparison. **D** Expression of Dl-NB2A results in a weaker ectopic induction of Wg expression, largely restricted to the dorsal compartment (arrow). The anterior stripe is missing due to increased CI. **E** Likewise, expression of Dl-CB2A also induces weaker ectopic expression of Wg than Dl, indicating that it is required for the full activity of Dl. Note that expression of Dl-CB2A leads to a stronger Wg activation than Dl-NB2A (longer stripe of ectopic expression), indicating that the CB is less important. **F** The mutation of both boxes results in an ectopic induction of Wg expression comparable to Dl-NB2A (compare with **D**), indicating that the NB is more important for Dl function. **G** Dl-N684 induced ectopic expression of Wg comparable to Dl-NB2A, indicating its importance for the function of the NB. **H** Expression of Dl-NB2A with re-introduced N684 causes a phenotype that is similar to that caused by Dl-NB2A (compare with **D**). **I** The mutation of the NN-motif in the ICD (Dl-DNNI2A) has no effect on the activity of Dl (arrows, compare with **B**). **J** Quantification of the CI and transactivation abilities of the Dl-variants shown in **C**–**I**. CI was measured by dividing the length of the gap of endogenous Wg expression by the total length of the endogenous Wg expression along the D/V boundary. Ectopic activation of the Notch pathway was determined by measuring the length of the anterior and/or posterior ectopic Wg expression and dividing it by the endogenous wg expression along the D/V boundary. ‘relative ectopic Wg’ displays the extend of ectopic Wg expression, while ‘% of CI’ shows the % of interruption of the endogenous Wg expression domain. Ten discs were measured for each genotype. *p* < 0.05 = *; *p* < 0.01 = **; *p* < 0.001 = ***

Previous work showed that bipartite binding of MIB1 (via its MZM and REP domains) to the NB and CB is required for the full activation of JAG1 [7] (Fig. 2A). However, the mutation of only the CB did not significantly affect MIB1-mediated JAG1/Notch signalling in cell culture experiments [7]. Moreover, previous experiments in *Drosophila* suggested that only the NB (ICD2) in Dl is required for Mib1-mediated Dl-signalling, although a sequence similar to the CB of JAG1, termed ICD3, is recognisable and conserved among insect Dl orthologs [6] (Figs. 1B, 2B and Additional file 1: Fig. S1). A caveat of the previous *Drosophila* work is that the conclusions were based on the analysis of random insertions of the Dl-variants in the genome. Thus, weak effects might have been missed due to the different expressivity of the constructs caused by position effects. In addition, the NB and CB were deleted in the tested variants. The deletions included strongly conserved Ks, which are important for the function of Dl because they might serve as ubiquitin adapters (see below). We therefore tested variants of Dl where the AAs of the NB, the predicted CB, or both boxes were replaced by alanine (A), but the conserved Ks were left in place. We found that the mutation of the NB (Dl-NB2A) resulted in a strong reduction of the activity of Dl expressed by *ptc*Gal4 in wildtype discs. This is indicated by the reduced ectopic expression of Wg in comparison to Dl expression shown in Fig. 2C, D, quantification in J: only one (posterior) stripe is present, which is also dramatically reduced in length and largely restricted to the dorsal compartment (Fig. 2D, arrow). This confirms the meaning of the NB/ICD2 [6, 7]. Nevertheless, also the mutation of the predicted CB/ICD3 reduced the activity of Dl, indicated by the loss of the anterior stripe of ectopic Wg expression upon Dl-CB2A expression (Fig. 2E, arrows). However, the loss of activity of Dl-CB2A was significantly weaker than that of Dl-NB2A, indicated by the longer remaining posterior stripe reaching also into the ventral compartment (Fig. 2E, arrows, compare with C, quantification in J). This indicates that the CB is less important for the activity of Dl. This is in agreement with the cell culture experiments performed with JAG1 [7]. We also generated variants where the Ks flanking the boxes are mutated to alanine. These variants showed similar phenotypes (see Additional file 1: Fig. S2, compare with Fig. 2D, E).

The combined mutation of both boxes caused a phenotype similar to the loss of only the NB, confirming that the NB is the prevailing binding epitope for Mib1 and that the CB largely depends on the presence of the NB (Fig. 2F, compare with D, quantification in J). This is in line with the previous cell culture experiments which showed that the reduction in activity of JAG1 signalling is similar, if the NB alone, or the NB and CB are mutated together [7]. It has been shown that a peptide containing the NB of Dl can bind to the MZM domain of MIB1 and that the deletion of the NB of Dl abolishes the binding to Mib1 in a co-immunoprecipitation assay [6, 7]. Combined with our results here, it suggests a scenario in which Mib1 would initially contact the Dl-ICD via binding to the NB with its MZM domain and then strengthen the association by binding to the CB with its REP domain. In this scenario, the CB is not sufficient for efficient Mib1 binding in the absence of the CB.

The quantification of the signalling activity and the extent of CI shown in Fig. 2J highlights the negative correlation between CI and signalling activity of the Dl variants, which we have previously also observed with the DlK2R variant [8, 16]. It further indicates that suppression of CI and signalling inversely depends on the presence of the NB and/or CB and therefore probably on the strength of the interaction with Mib1: strong interaction with Mib1 results in strong signalling and weak CI, while weak interaction decreases signalling and increases CI. Importantly the quantification also revealed that DlK2R is less active than Dl-NB + CB2A, since DlK2R displays less signalling activity, but tends to have more CI (Fig. 2J).

### N684 of the N-box is important for the interaction of Dl with Mib1

The determined atomic structure suggests that in the NB of JAG1, a centrally located asparagine (N1124) is pivotal in the interaction with the MZM domain of MIB1 [7]. The corresponding asparagine in the NB of Dl is N684 (Fig. 2B, arrow). To determine the importance of N684 for the Mib1-induced activity of Dl in vivo, we replaced it by alanine. We found that the activity of Dl-N684A is similar to that of Dl-NB2A, indicating its importance for function of the NB (Fig. 2G, compare with D, arrows, quantification in J). However, the individual re-introduction of N684 in Dl-NB2A fails to increase the activity of Dl-NB2A, indicating that, although N684 is essential, other AAs in the N-box contribute to the binding of Dl to Mib1 (Fig. 2H, compare with D, quantification in J). This indicates the NB of Dl with the MZM domain of Mib1 in *Drosophila* follows similar rules than that previously found for that between JAG1 and MIB1. Altogether, the results indicate that, similar to JAG1, Dl possesses similar functional N- and C-boxes, which are both required for its full activity. They also indicate that the NB is the prevailing binding epitope for Mib1-induced Dl-signalling.

Analysis of the ICD of the Dl-ortholog DeltaD of the zebrafish (*Danio rerio*) identified an additional conserved di-asparagine (NN) motif which is relevant for enhancing the signalling by the NB [23]. A similar motif is present in Dl orthologs of several insect species, although located more C-terminally [6]. We mutated the corresponding VNNI sequence to A to test its significance for the function of Dl. We found that this change had no detectable effect on the signalling activity of Dl (Dl-VNNI2A, Fig. 2I, compare with C, quantification in J). Thus, the NN-motif appears to be dispensable for Mib1-mediated activation of Dl.

### The importance of the NB and CB of Dl for the development of *Drosophila*

To further confirm the function of the NB and CB of Dl at the organismal level, we generated *Dl*-knock-in alleles, which encode variants with the NB, or CB, or both mutated to A. For this purpose, we used the *Dl*^*attP*^-allele where most of the Dl coding sequence (exon 6) is replaced by an attP-landing site to generate knock-in alleles encoding Dl-variants with mutated NB, CB and both boxes [24]. As in the constructs used for Gal4 over-expression experiments, the Ks flanking the binding domains were left in place.

**Fig. 3:**
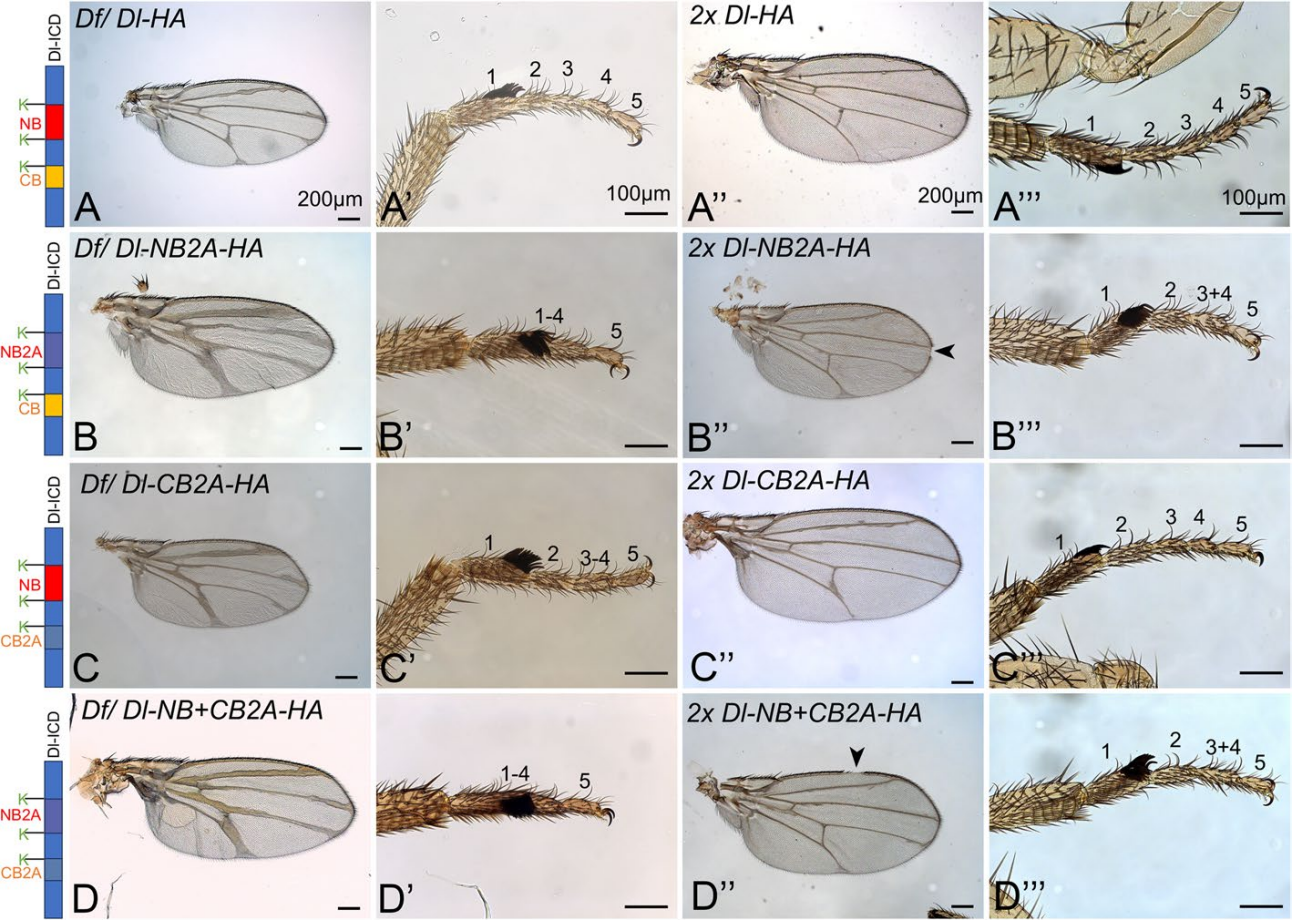
Adult phenotypes of Dl^attP^-knock-in alleles encoding Dl-variants with NB, CB or NB + CB mutated over the deficiency Df(3R) Dl^BSC850^ (Df) and in homozygosity. All variants contain the Ks flanking the boxes, to exclude effects caused by the absence of crucial ubi acceptors. **A**, **A’** Dl-HA/Df results in a haplo-insufficient phenotype with strong broadening of wing veins. The tarsal region consisting of 5 segments has a wildtype appearance. **A’’**, **A’’’** Two copies of Dl-HA restore the WT phenotype of the wing and leg. **B**, **B’** The phenotype of one copy of Dl-NB2A/Df flies. The wing veins are broadened (**B**) and the tarsal segments 1–4 are fused (**B’**). **B’’**, **B’’’** Phenotype of homozygous Dl-NB2A flies. The wing vein broadening and tarsal segment fusion phenotypes are less severe. The arrowhead points to a small Notch in the wing margin. **C**, **C**’ The mutation of the CB causes a milder vein and leg phenotype. **C’’**, **C’’’** In homozygosity of Dl-CB2A, only weak wing and leg defects. **D**, **D’** Simultaneous mutation of the NB and CB causes developmental defects similar to Dl-NB2A (compare with **B**–**B**’’’). The arrowhead points to a small notch in the wing margin

We first tested whether the addition of the HA-tag (which does not contain Ks) to the C-terminus had an effect on the activity of Dl (Additional file 1: Fig. S3). For this purpose, we compared the phenotype of *Dl*^*attP*^*-HA-Dl*, a variant with the HA inserted in the extracellular domain close to the membrane, with our previously generated C-terminally tagged *Dl*^*attP*^*-Dl-HA* [16]. We asked whether the presence of just one copy of the *Dl*-alleles in the genome can rescue *Drosophila* development, as has been observed for the previously characterised *Dl*^*attP*^*-Dl-HA* and *Dl*^*attP*^*-DlK2R-HA* [16]. For this purpose, we monitored the phenotype of the alleles over a chromosomal deficiency of *Dl*, *Df(3R) Dl*^*BSC850*^ (*Df*). Both differently tagged Dl-variants, *Dl*^*attP*^*-HA-Dl* and *Dl*^*attP*^*-Dl-HA*, rescued the *Dl*-mutant neurogenic phenotype in a comparable manner, allowing the development to the adult stage. As expected for fully functional alleles, the flies displayed the characteristic haplo-insufficient phenotype of *Dl* (Additional file 1: Fig. S3A–B’, compare with Fig. 3A, A’). This indicates that the addition of the HA-tag at the C-terminus does not significantly affect the activity of Dl.

Having confirmed the functionality of the C-terminal HA-tagged Dl^attP^-variant, we analysed the activity of *Dl*^*attP*^*-Dl-NB2A-HA*, *Dl*^*attP*^*-Dl-CB2A-HA* and *Dl*^*attP*^*-Dl-NB* + *CB2A-HA*. We found that already one copy over the deficiency provided sufficient activity to prevent the embryonic neurogenic phenotype characteristic for *Dl* mutants and allowed the development to the adult stage in all cases (Fig. 3). However, the phenotypes of the alleles differed and were stronger than the haplo-insufficient *Dl*-phenotype (compare Fig. 3A, A’, B, B’, C, C’). The phenotype of *Dl*^*attP*^*-Dl-NB2A-HA/Df* was stronger than that of *Dl*^*attP*^*-Dl-CB2A-HA/Df*, indicated by the stronger broadening of the wing veins and fusion of more tarsal segments (Fig. 3B, B’, C, C’). Moreover, the phenotype of *Dl*^*attP*^*-Dl-NB* + *CB2A-HA/Df* flies was comparable to that of *Dl*^*attP*^*-Dl-NB2A-HA/Df*, confirming the prime importance of the NB for Mib1-dependent Dl-signalling, which was also found in the over-expression experiments (compare Fig. 3D, D’ with B, B’).

As previously reported, homozygous *Dl*^*attP*^*-Dl-HA* flies have a wildtype appearance, indicating that it is a fully functional (wildtype) allele [16] (Fig. 3A’’–A’’’). In contrast, homozygosity of *Dl*^*attP*^*-DlK2R* leads to a stronger phenotype due to its stronger cis-inhibitory abilities [16]. The phenotype of *Dl*^*attP*^*-Dl-NB* + *CB2A-HA*, *Dl*^*attP*^*-Dl-NB2A-HA* and *Dl*^*attP*^*-Dl-CB2A-HA* weakened in homozygosity, indicating that they are not as cis-inhibitory than DlK2R-HA (Fig. 3B–D’’’). Nevertheless, the phenotype of homozygous *Dl*^*attP*^*-Dl-CB2A-HA* flies was weaker than that of *Dl*^*attP*^*-Dl-NB2A-HA* and *Dl*^*attP*^*-Dl-NB* + *CB2A-HA*, while the phenotype of homozygous *Dl*^*attP*^*-Dl-NB2A-HA* and *Dl*^*attP*^*-Dl-NB* + *CB2A-HA* flies was very similar, again highlighting the importance of the NB (compare Fig. 3C’’, C’’’ with B’’, B’’’ and D’’ and D’’’).

### Transport of Dl to the plasma membrane does not depend on its ICD

**Fig. 4:**
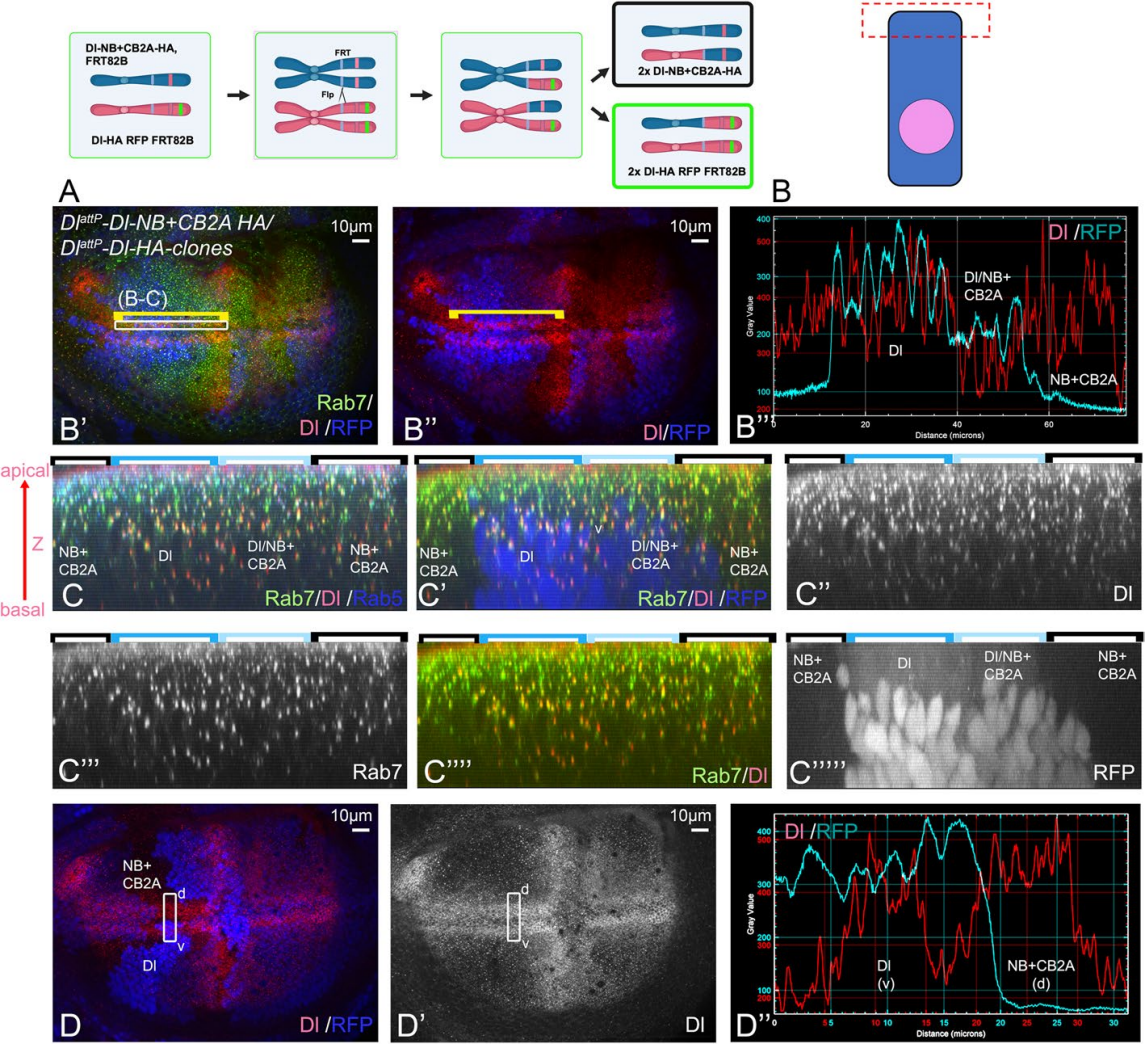
Comparison of the endocytic behaviour of Dl-NB + CB2A-HA with DlK2R-HA. **A** Cartoon describing the generation of the adjacently located homozygous clones by Flp/FRT-mediated clonal analysis (mitotic recombination). The mitotic recombination in G2 results in the free segregation of the replicated mutant alleles because of their localisation on different centromeres. The result is homozygous founder cells for each genotype in 25% of the cases. **B**–**B’’** Comparison of the apical membrane localisation of the Dl-variants in cells of clones homozygous for Dl^attP^-Dl-NB + CB2A-HA (absence of RFP) and Dl^attP^-Dl-HA (homozygous for RFP) detected with a Dl antibody directed against the extracellular domain. **B’’’** Pixel intensity plot of the RFP channel which identifies the clones and the Dl channel. The yellow bracket highlight the region of measurement adjacent to the D/V-boundary where Dl is expressed in a continuous stripe from anterior to posterior at the same level in wildtype discs. It includes regions homozygous for both Dl alleles. No significant difference is observed in the apical levels of Dl in Dl^attP^-Dl-NB + CB2A-HA and Dl^attP^-Dl-HA homozygous cells, indicating that both Dl variant are present in the apical membrane at comparable levels. **C**–**C’’’’** Z section of the region of Dl expression highlight in **B’** with the yellow bracket. It reveals that intracellular Dl and Dl-NB + CB2A are located in Rab7- and Rab5-positive endosomes. **D**–**D’’** Another example of the correct location of and levels of Dl-NB + CB2A in the apical membrane. Here the Dl-homozygous clone is located in the ventral stripe of Dl and the Dl-NB + CB2A-homozygous clone in the dorsal stripe. The pixel density plot shown in **D’’** confirms the similarity of the levels of the two Dl-variants in the apical membrane domain of the cells

We were interested in the general importance of ICD of Dl at the organismal level. Therefore, we generated and analysed the knock-in allele *Dl*^*attP*^*-DlΔICD-HA*, which encodes a variant that lacks most of its ICD (12 amino acids long ICD, the Ks are replaced by Rs; see Additional file 1: Fig. S4). In addition, the lysines in its residual ICD are exchanged to arginines. As expected, this allele is not functional, indicated by its lethality in homozygosity. However, anti-HA-staining of heterozygous wing discs revealed that *Dl*^*attP*^*-DlΔICD-HA* was expressed in the correct pattern (Additional file 1: Fig. S4A, A’). The comparison with Dl::GFP, a genome edited fully functional allele of *Dl* [25], also showed that it was present at the apical membrane together with Dl::GFP (Additional file 1: Fig. S4B–C’’, white arrow in C’, C’’). In addition, it was also mis-localised in the baso-lateral membrane domain, where Dl::GFP was largely absent (Additional file 1: Fig. S4C’’, arrowhead). This finding shows that the transport of Dl to the plasma membrane (exocytosis) does not require its ICD. Importantly, DlΔICD-HA was basically absent from Dl::GFP positive intracellular punctae (Additional file 1: Fig. S4C–C’’, red arrow). These punctae were identified to be endosomes [8, 26, 27] (Fig. 4C–C’’’’’). Combined, these findings indicate that endocytosis of DlΔICD-HA is strongly impaired. The findings are in agreements with previous reports that show that a similar Dl-variant without ICD, Dl^ΔC^, accumulates in the membrane as a result of impaired endocytosis [20, 26]. Thus, the ICD of Dl is important for efficient endocytosis, but not exocytosis.

The previous work also indicated that Dl^ΔC^ acts in a dominant-negative fashion upon over-expression [20, 26]. Therefore, we were surprised to obtain the *Dl*^*attP*^*-DlΔICD-HA* allele, since we expected that its expression, albeit at the endogenous level, would lead to lethality in heterozygosity and therefore of the initial transformants. Indeed, our clonal analysis revealed that *Dl*^*attP*^*-DlΔICD-HA* is cell lethal, indicated by the presence of only wildtype orphan clones (Additional file 1: Fig. S4D, D’, arrows). Moreover, we found that DlΔICD-HA/Dl-A cells accumulated higher HA-levels in the apical plasma membrane than homozygous Dl-HA cells (Additional file 1: Fig. S4 D’’–F). In order to further investigate the activity of *Dl*^*attP*^*-DlΔICD-HA*, we ectopically expressed the glycosyltransferase Fringe by *ptc*Gal4. In the wildtype, this expression results in the ectopic activation of the Notch pathway at its posterior expression boundary, indicated by the ectopic expression of Wg (Additional file 1: Fig. S4G). The induction of the ectopic activity of the Notch-pathway requires the signalling of both ligands, Dl and Ser [12, 28, 29]. However, it is initiated by Dl, also indicated by the finding that the length of the ectopic stripe of Wg is reduced in Dl-heterozygous compared to wildtype discs (Additional file 1: Fig. S4G, G’, arrow, quantification in H) [29]. We found that this stripe is further shortened in discs with one wildtype copy and one copy of *Dl*^*attP*^*-DlΔICD-HA* (Additional file 1: Fig. S41G’’, arrow, quantification in H). This result suggests that the presence of *Dl*^*attP*^*-DlΔICD-HA* has a negative effect on the activation of the Notch pathway by the remaining copy of wildtype Dl, as it is expected from a dominant-negative acting variant. It appears that the presence of the wildtype copy in *Dl*^*attP*^*-DlΔICD-HA/* + flies can buffer the dominant-negative effect of *Dl*^*attP*^*-DlΔICD-HA*. This buffering effect allows the heterozygous *Dl*^*attP*^*-DlΔICD-HA/* + flies to survive.

### Endocytosis of Dl-NB + CB2A is not significantly affected

We here and previously found that the ability of Dl to cis-inhibit is inversely correlated with its endocytosis efficiency [16]. To investigate the efficiency of endocytosis of Dl^attP^-Dl-NB + CB2A-HA relative to Dl and DlK2R, we used clonal analysis to generate cell clones that only express Dl^attP^-Dl-NB + CB2A-HA adjacent to clones that express either only Dl^attP^-Dl-HA (Fig. 4A). This enabled us to directly compare the subcellular localisation of the variants in wing disc cells. We previously reported that clones homozygous for *Dl*^*attP*^*-Dl-HA* and clones homozygous for endogenous wildtype Dl displayed comparable levels of Dl at their surface, indicating that *Dl*^*attP*^*-Dl-HA* behaves as a wildtype allele [16].

Dl is located in the apical membrane in the imaginal disc cells. In the twin clone assay, we observed no significant increase in the apical membrane levels of Dl^attP^-Dl-NB + CB2A + K-HA compared to Dl^attP^-Dl-HA (Fig. 4A–B’’’). Moreover, Dl^attP^-Dl-NB + CB2A + K-HA was present in most of the Rab7-positive endosomes in a similar frequency as Dl-HA (Fig. 4C–C’’’’). Combined with the observation that the ICD of Dl is not required for exocytosis, this result suggests that Dl-NB + CB2A is endocytosed with a similar efficiency than Dl^attP^-Dl-HA. The previous comparison of homozygous *Dl*^*attP*^*-Dl-HA* with homozygous *Dl*^*attP*^*-DlK2R-HA* clones revealed that Dl^attP^-DlK2R-HA accumulated to a much higher level in the plasma membrane than Dl [16]. Thus, it appears that, just like Dl, Dl-NB + CB2A-HA is more efficiently endocytosed than DlK2R-HA. These results are in agreements with the finding that loss of *mib1* function hardly affects the endocytosis of Dl [11, 27]. The difference in endocytosis efficiency between Dl-NB + CB2A-HA, which is deficient for Mib1-binding, and DlK2R-HA, which cannot be ubiquitylated, supports the previously drawn conclusion that ubi of Dl is not solely mediated by Mib1, but also by one or more unidentified E3-ligase(s) [16].

We have previously shown that the efficiency in endocytosis is crucial for the proper activity of Dl. Its decrease results in an increase in CI and a decrease in signalling [8, 16]. Thus, we conclude that the higher efficiency in endocytosis of Dl-NB + CB2A is the likely cause of its slightly weaker CI and stronger signalling activity compared to DlK2R (see quantification in Figs. 1H and 2J).

### The importance of the MZM and REP domains for the function of Mib1

To further investigate the interaction of Dl with Mib1, we generated Mib1-variants lacking either the REP domain alone or the MZM and REP domains together (Additional file 1: Fig. S5). We expressed the generated variants under control of the tubulin promoter (tubP) to achieve ubiquitous expression at natural abundance, as observed for endogenous Mib1 in the wing disc [11]. All variants are inserted into the same landing site to achieve comparable expression. We tried to generate transgenic flies bearing a *tub.P-mib1* insertion with the MZM domain deleted (*tubP-mib1ΔMZM*), but failed to obtain insertions that could be maintained as a stock, although we tried to obtain transformants several times. Although buffered by the presence of two endogenous copies of wildtype *mib1*, the initially transformed flies, which carried just one copy of *tubP-mib1ΔMZM* in their genome, displayed many *Notch*-related defects, were nearly sterile and survived only for a short time, which made their maintenance as a stock and analysis impossible. Thus, it appears that *tubP-mib1ΔMZM* acts in a dominant-negative manner. This dominant-negative activity depends on the presence of the REP domain, since we obtained transformants for a variant that lack both domains (*tubP-mib1ΔMZM* + *REP*), or only the REP domain (*tubP-mib1ΔREP*) without problems. It appears that the MZM domain somehow suppresses a deleterious activity of the REP domain.

**Fig. 5:**
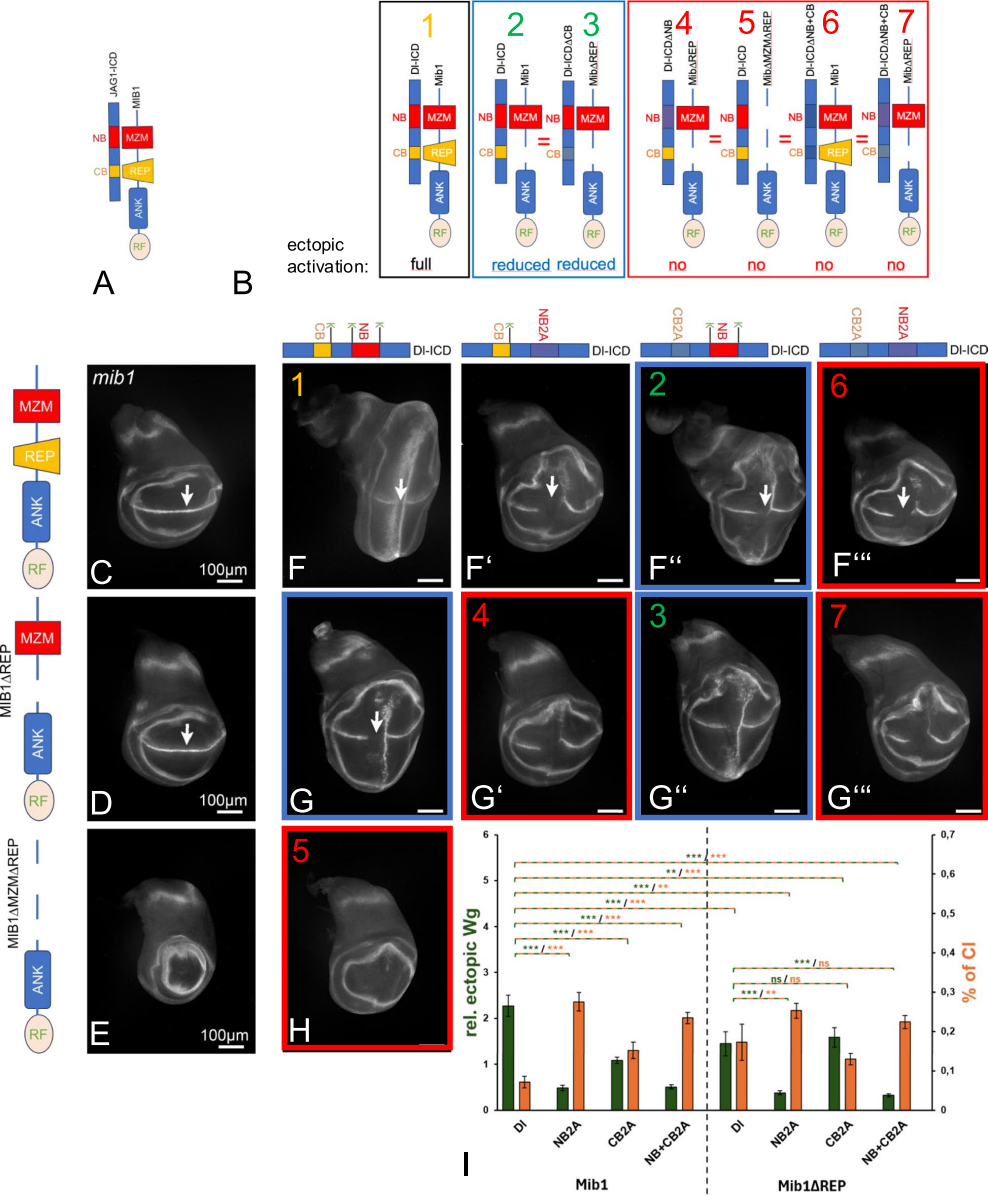
The MZM and REP domains of Mib1 interact with the NB and CB of Dl, respectively. **A** Reported interaction between JAG1 and MIB1. The NB and CB of JAG1 bind to the MZM and REP domains of MIB1, respectively. **B** Prediction for the ectopic activation of Notch by combinations of Dl- and Mib1-variants in mib1 mutant discs. Note that the combination of Dl-NB2A with Mib1ΔREP is predicted not to function, since the interaction of both the NB with the MZM and the CB with the REP should be compromised. It should therefore cause a similar phenotype as the other combinations in the red frame. However, the combination of Mib1ΔREP with Dl-CB2A should lead to the reduction of ectopic activation in a similar manner like the combination of Dl with Mib1ΔREP (combinations in the blue frame. **C**–**E** Rescue of mib1 mutant flies with Mib1-variants expressed under control of tubP. Rescue is performed with one copy of the constructs. **C** tubP-mib1 completely rescued mib1 mutants and the expression of Wg along the D/V boundary is re-installed (arrow, compare with Fig. 1E). **D** Likewise, tubP-mib1ΔREP resulted in a re-instalment of Wg expression along the D/V-boundary (arrow). **E** In contrast, tubP-mib1ΔMZM + REP failed to rescue mib1 mutants. The disc resembled that of mib1 mutant discs (compare with Fig. 1E). **F**–**H’’’** Combinations of Mib1- and Dl-variants expressed in mib1 mutant discs. The Mib1 variants are expressed under control of tubP; the Dl-variants are expressed with ptcGal4. The coloured frames highlight the results, which confirmed the predictions outlined in **B**. **I** Quantification of the signalling activities and CI of the combinations

Already the presence of one copy of the full-length variant *tubP-mib1* resulted in a complete rescue of *mib1* null-mutants (Fig. 5A, C, Additional file 1: Fig. S5A–D’). As expected from previous work [7, 10, 30, 31], the presence of *tubP-mib1ΔMZM* + *REP*, which lacks both ligand interaction domains, failed to rescue *mib1* mutants, indicating that Mib1ΔMZM + REP has no activity and confirming the importance of the MZM and REP domains for Mib1 function and ligand activity (Fig. 5E, Additional file 1: Fig. S5C, D, C’’’, D’’’). Interestingly, also the presence of one copy of *tubP-mib1ΔREP* in the genome caused a nearly complete rescue of *mib1* mutant discs, indicated by the grossly normal expression of the Notch target gene Wg along the dorso-ventral boundary of wing discs and the grossly normal wings and legs of the imago (Fig. 5D, arrow, Fig. 5S1C’’, D’’). The *tubP-mib1ΔREP* rescued adults had no defects with the exception of a small patch of extra vein at the tip of wing vein 2 (Additional file 1: Fig. S5C’’, arrow). Thus, the MZM domain mediates most of the activity of Mib1.

### Interaction of the NB and CB of Dl with Mib1

MIB1 binds via its MZM and REP domains to the NB and CB of JAG1 (Fig. 5A). In addition, the MZM domain of MIB1 can also bind to a peptide containing the NB of Dl [7]. Having demonstrated the functionality of the predicted CB of Dl, we tested whether Dl also uses the bipartite interaction mode to bind to Mib1. To do so, we co-expressed combinations of the generated Dl-NB and -CB variants (with flanking Ks mutated) with the *tub.mib1*-deletion-variants in *mib1* mutants and assayed the activity of the Notch pathway. As expected, the activity of the Dl-variants was dramatically suppressed in *mib1* mutants (Additional file 1: Fig. S5E–G). The sole expression of Dl, Dl-NB2A and Dl-CB2A in *mib1* discs rescued by *tub.mib1* resulted in the activation of the Notch pathway similar to expression of the Dl-variants in wildtype discs, confirming that one copy of full-length Mib1 can provide the full function (Fig. 5F–F’’’, compare with Fig. 2C–F).

If Dl interacts with Mib1 in the bipartite binding mode, clear predictions should be fulfilled which are outlined in Fig. 5B. The main predictions are 1. The combination of Dl-NB2A with Mib1ΔREP should not lead to ectopic activation of Notch, since both of the bipartite interactions are compromised. Hence, it should produce the same phenotypes as the combinations of Dl with Mib1ΔMZMΔREP and Dl-NB + CB2A with Mib1. These predictions were fulfilled by our experiments (see Fig. 5G’, H, F’’’, respectively, framed in red, quantification in J). The second main prediction is that the combination of Dl-CB2A should produce the same reduced ectopic activation of the Notch-pathway if combined with Mib1 or Mib1ΔREP than the lack of the CB in Dl makes the presence of the REP domain in Mib1 unnecessary. Also, this prediction is fulfilled as seen in Fig. 5G and G’’, respectively, framed in blue, quantification in J. In addition, the predictions of the model for the activity produced by the other tested combinations are fulfilled by the experiments (Fig. 5B, F–H, quantification in J). Altogether, the results strongly suggest that Dl binds to Mib1 via the interaction of its NB with the MZM domain and its CB with the REP domain in vivo. Thus, it appears that the interaction between Dl and Mib1 follows similar rules as previously found for the binding of JAG1 to MIB1 and therefore are of general importance.

Note that CI of Dl is enhanced in *tubP-mib1ΔREP* rescued *mib1* mutant discs, as previously found for the loss of the Ks in the ICD of Dl (Fig. 5D, E, arrow, quantification in J). Moreover, in discs rescued by full-length Mib1, the Dl-variants that lack one of the binding boxes are more cis-inhibitory than Dl (compare Fig. 5F with F’–F’’’, arrows, I). This observation further supports the notion that the strength of binding of Mib1 to Dl and the presence of the Ks are required to suppresses/adjust CI and signalling.

### Identification of the Ks in the ICD of Dl important for signalling

**Fig. 6:**
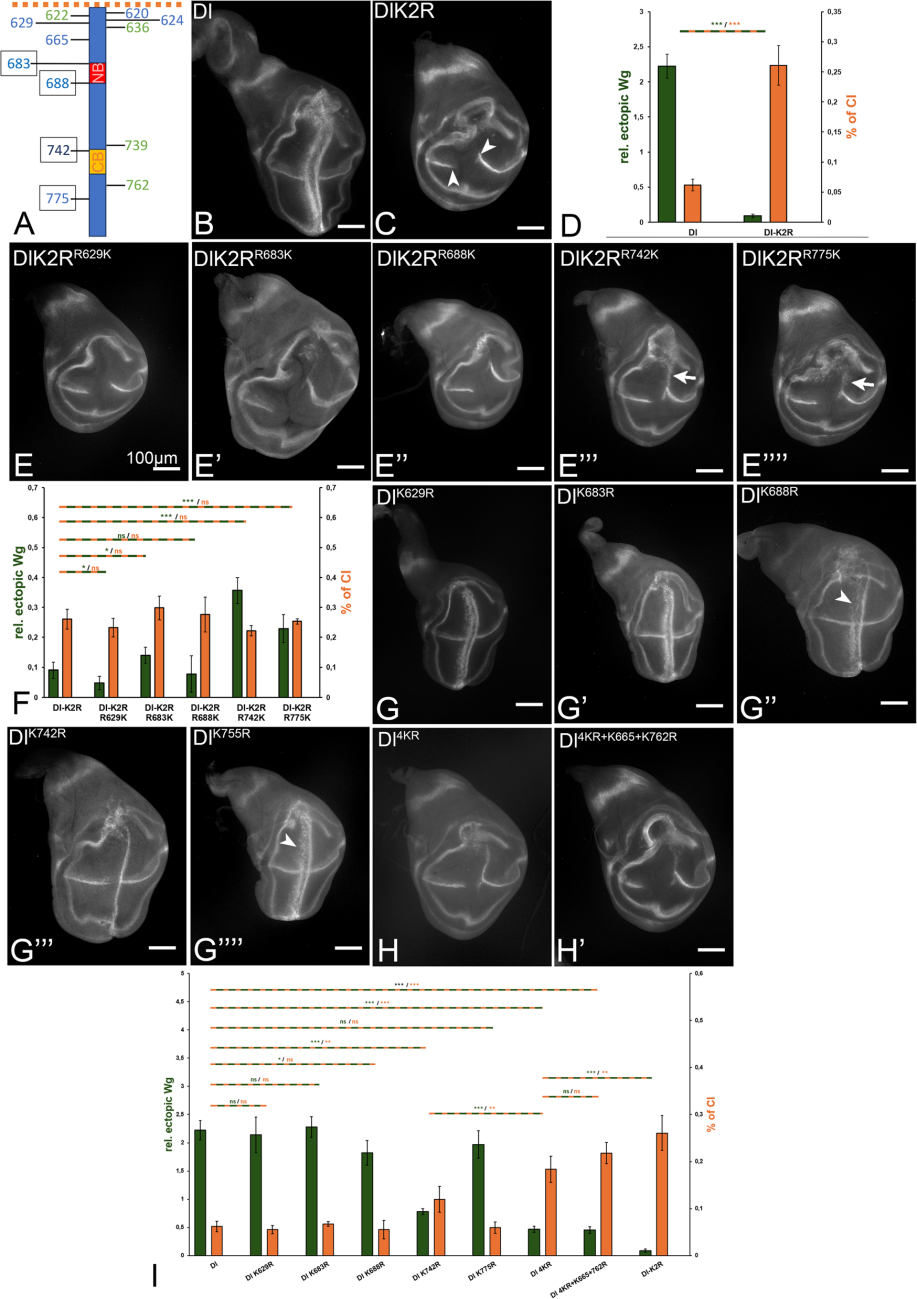
Identification of Ks in the ICD of Dl relevant for signalling. See also Fig. 6S1 for a summary of all variants tested. **A** Location of the 12 Ks in the ICD of Ser of *Drosophila*. **B**, **C** Consequences of expression of Dl and DlK2R for comparison. **D** Quantification of the expression of Dl and DlK2R. **E**–**E’’’’** Consequences of the re-introduction of individual core Ks into DlK2R. Quantification in **F**. **G**, **G’** Consequences of the exchange of individual core Ks to R into Dl. Quantification in **I**. **H**, **H’** Exchange of all 4 core Ks (**H**) or the 4 core Ks and K665 and K762 to R. Quantification in **I**. For further information, see text

**Fig. 7:**
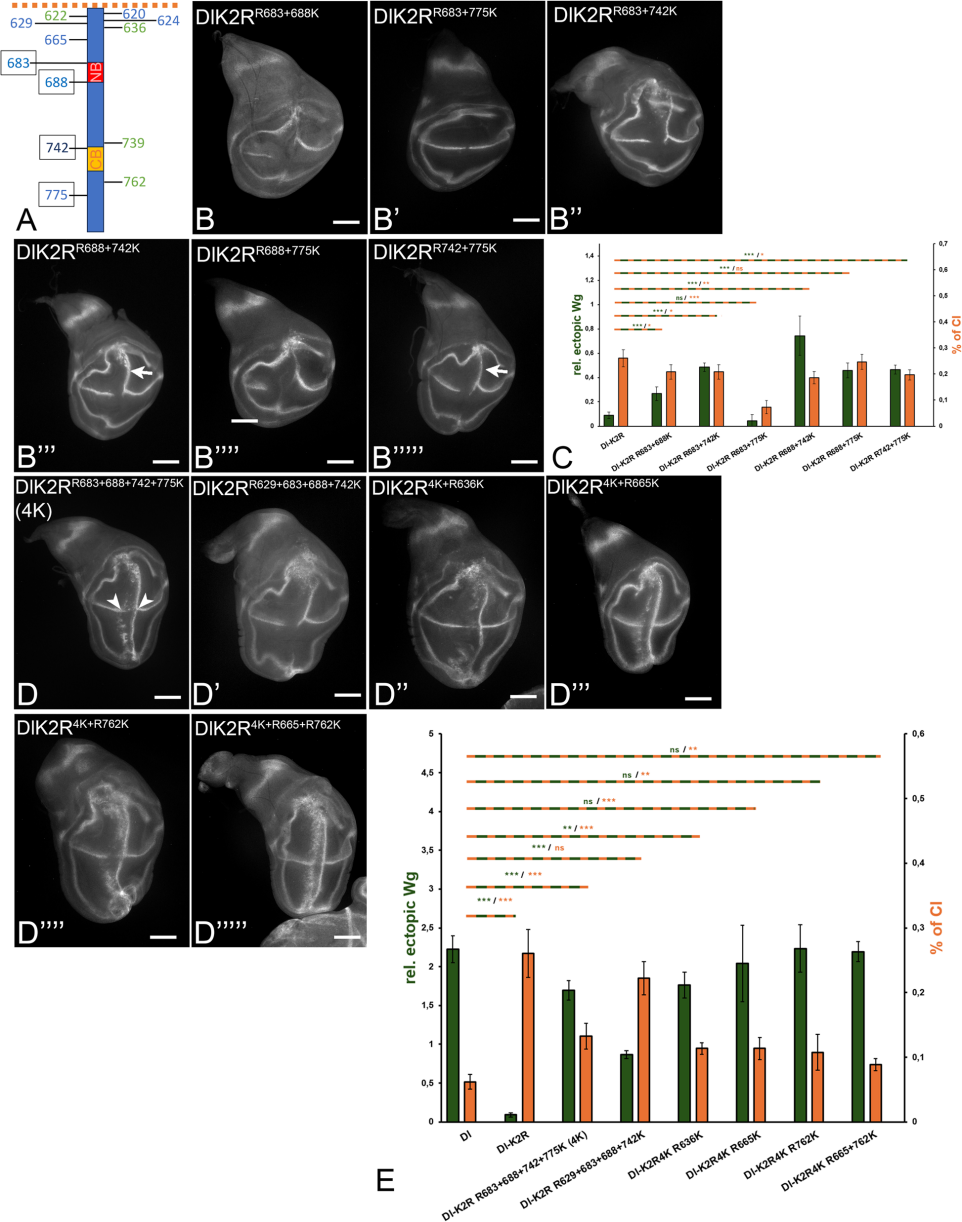
Identification of combinations of Ks in the ICD of Dl relevant for signalling. See also Fig. 6S1 for a summary of all variants tested. **A** Location of the 12 Ks in the ICD of Ser of *Drosophila*. **B**, **C** Consequences of expression of Dl and DlK2R for comparison. **B**–**B’’’’’** The phenotype of expression of DlK2R-variants where combinations of two core Ks were re-introduced. **C** Quantification of the activity of the Dl-variants shown in **B**–**B’’’’’**. **D**–**D’’’’’** Identification of the combination of Ks required for dl signalling. **E** Quantification of the activity of the Dl-variants shown in **D**–**D’’’’’**. It reveals the re-introduction of a combination of six Ks, including the core Ks, re-establishes the full activity of Dl, but is still insufficient to completely normalise CI. For further information, see text

Despite the ability of DlK2R to provide sufficient activity for complete development of *Drosophila*, the previous analysis also clearly showed that at least some of the 12 Ks in the ICD are important for the full activity of Dl in Mib1-dependent signalling events and also for adjustment of CI [8, 16]. We wondered which and how many of the Ks are important. To determine the importance of the conserved Ks, we performed two complementary sets of experiments: (A) we re-introduced individual Ks or combinations of them into DlK2R-HA and (B) replaced individual or combinations of Ks in Dl-HA by the similar R in Dl-HA (Fig. 6). The highlights of the analysis are summarised in Figs. 6 and 7; the complete results comprising all tested constructs are shown in Additional file 1: Fig. S6.

A comparison of the sequences of Dl-orthologs of insects revealed that 8 of the 12 Ks of Dl are conserved in other species to different degrees [6] (Additional file 1: Fig. S1, arrows, Fig. 6A). Three of the 8 conserved Ks are close to the transmembrane domain and together with adjacent Rs are probably required to stabilise the membrane integration of Dl by providing a positive charge that anchors the transmembrane domain in the plasma membrane. Indeed, these three Ks had no importance for the signalling activity of Dl, as their combined exchange to R in Dl, or re-introduction into DlK2R had no effect (DlK2R^R620+622+624 K^ and Dl^K620+622+624R^, Additional file 1: Fig. S7, also labelled with TM in Additional file 1: Fig. S6). Thus, they are not required for signalling. Of the remaining five, K683, K688, K742 and K775 are most conserved and we concentrated on these four Ks (core Ks). K683 and K688 are located at the edges of the NB and K742 at the N-terminal edge of the CB (Fig. 6A).

#### Re-introduction of the core Ks into DlK2R

The individual re-introduction of two Ks, K742 and K775 (DlK2R^R742K^ and DlK2R^R775K^), had a stronger positive effect on the activity of DlK2R. They induced one posterior stripe of ectopic Wg expression largely restricted to the dorsal compartment (Fig. 6E’’’, E’’’’, arrow, compare with C). Introduction of K742 induced a significantly longer stripe of ectopic Wg expression than K775, suggesting that it is more important for function (compare Fig. 6E’’’ with E’’’’, arrow, quantification in F). Nevertheless, DlK2R^K742^ and DlK2R^R775K^ displayed only weak activity compared to Dl (compare Fig. 6B with E’’’ and E’’’’). The individual re-introduction of the other two core Ks into DlK2R had only marginal effects on signalling (Fig. 6E’, E’’, compare with C, quantification in F). These findings suggest that K742 is the most important K in the ICD of Dl, followed by K775, K683 and K688.

The results confirmed that more than one K is required for the full function of Dl. We therefore tested combinations of two, three and four core Ks introduced into DlK2R (Fig. 7 and Additional file 1: Fig. S6). This analysis showed that 1. The re-introduction of K683 or K688 had a weak enhancing effect, only if combined with K742, indicated by the stronger stripe of ectopic Wg expression (Fig. 7B–B’’’, quantification in C). The enhancing effect of K683 and K688 on K742 was similar to the combination of K742 with K775 (Fig. 7B’’, B’’’ arrow, compare with B’’’’, quantification in C). Thus, the two Ks contribute to the activity of Dl, but only in combination with K742 and/or K775. In the case of the re-introduction of combinations of three Ks, the ones with K742 were the most active ones, indicated by the longer stripes of ectopic Wg expression (Fig. 6S1). 2. Although the re-introduction of all four core Ks into DlK2R (DlK2R^4K^) resulted in strong activity, it did not completely restore the activity of Dl, since the anterior stripe of ectopic Wg expression was not continuous as observed in Dl (Fig. 7D, compare with Fig. 6B, quantification in 7E). This result indicates that less conserved Ks in addition to the core Ks are required for the full activity of Dl. Note that the re-introduction of the core Ks into the ICD of DlK2R reduces the gap in the expression of endogenous Wg along the D/V boundary compared to DlK2R, indicating a reduction in CI (Fig. 7D, compare with Fig. 6C, quantification in E). Thus, the core Ks, which are important for signalling, are also important for the suppression/adjustment of CI of Dl. The finding highlights the tight inverse correlation between the ability Dl to signal and to cis-inhibit in Mib1-dependent processes. The replacement of K775 of the core by K629 (DlK2R^R629+683+688+742 K^) resulted in a reduction of the activity (Fig. 7D’, quantification in E). This suggests that the presence of all core Ks is required for the full activity of Dl.

#### Exchange of the conserved Ks by Rs in Dl

The replacement of K742 by R reduced, but not abolished the activity of Dl. In this case, the anterior ectopic stripe of Wg expression was lost (Fig. 6G’’’, compare with Fig. 6B, quantification in I). A very mild reduction was also observed in the case of the individual replacement of K688 (Fig. 6G’’). In this case, the anterior stripe of expression of Wg was slightly reduced in the dorsal compartment in comparison to Dl-HA expression (arrowheads in Fig. 6G’’, compare with Fig. 6B, quantification in I). The exchange of K683 and surprisingly K755 did not lead to a recognisable reduction (Fig. 6G’, G’’’’, quantification in I). Even the replacement of all 4 core Ks by R did not completely abolish the activity of Dl (Fig. 6S1).

Altogether, the combined analysis revealed that the 4 core Ks are important for Mib1-mediated signalling of Dl, but differ in their importance. K742 is the most important one, followed by K775 and then by K688 and K683. The results also revealed that a combination of more than the 4 core Ks is required for the full function of Dl. The additionally required Ks are less conserved among the Dl orthologs of insects. Note that the replacement of the core Ks by Rs increases the gap in the endogenous expression of Wg along the D/V-boundary increases, indicating an increase in CI (Fig. 6S1). Combined with the observation that the addition of Ks into DlK2R reduces CI, the findings confirm that the Ks are involved in adjusting the degree of CI and signalling by Mib1.

### A combination of more than six Ks in the ICD restores the signalling activity of Dl

To identify additional Ks important for the activity of Dl, we tested less conserved Ks in concert with the core Ks (Fig. 6S1). Eventually, we identified K665 and K762 as additionally required Ks. Their re-introduction into DlK2R in addition to the four core Ks led to a signalling activity comparable to Dl (Fig. 7D’’’’’, compare with 6B, quantification in E). Nevertheless, DlK2R4K R665 + 762 K was still slightly more cis-inhibitory, indicating that additional Ks might be required for the re-establishment of the normal level of CI of Dl. Likewise, the replacement of these 6 Ks by Rs reduced the activity of Dl to a level close to DlK2R (Fig. 6H’, compare with C, quantification in I). Note that the addition of the Ks located at the transmembrane region to the 4 core Ks in DlK2R did not improve the activity of Dl. The same is true if K636 or 739 was added to the core Ks (see Fig. 6S1). This indicates that there is some specificity for K665 and K762 and that ubi by Mib1 is selective.

### The importance of the core Ks of the ICD of Dl for development

**Fig. 8:**
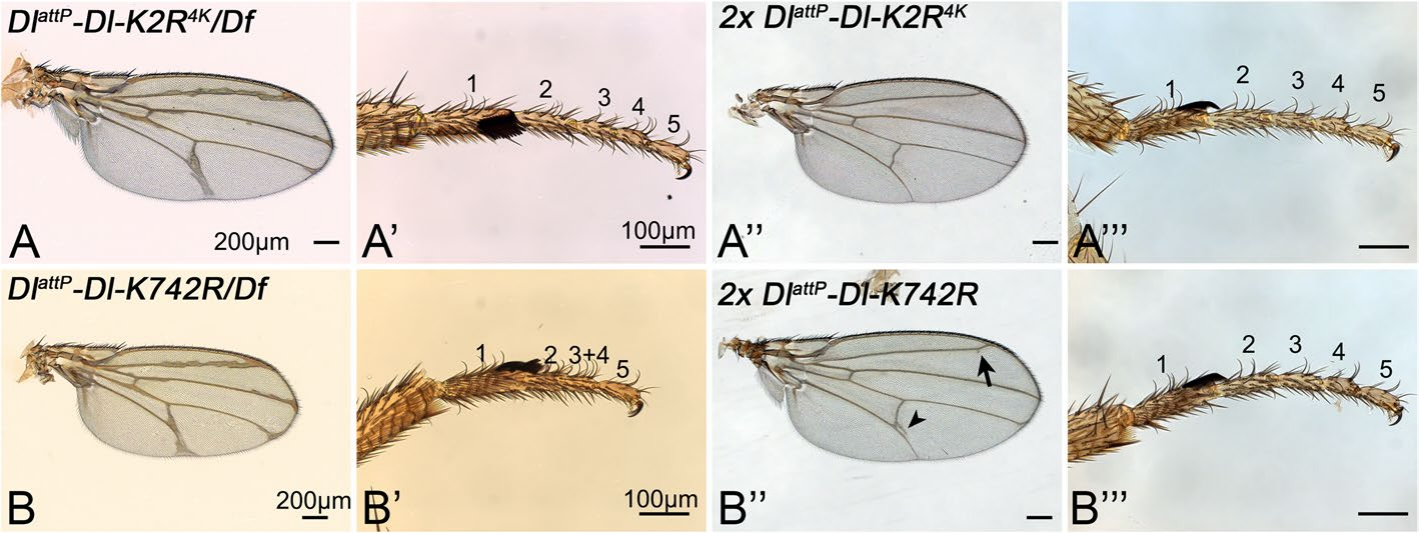
The importance of the four core Ks of the ICD off Dl. **A**–**A’’’** Dl^attP^-DlK2R^4K^ is a functional Dl allele. The phenotype of Dl^attP^-DlK2R^4K^ over the deficiency resembles that of Dl^attP^-Dl (compare with Fig. 3A, A**’**). **A**, **A’** Heterozygous Dl^attP^-DlK2R^4K^ flies display the phenotype typical for Dl heterozygosity (compare with Fig. 3A). **A’’**, **A’’’** Homozygosity of Dl^attP^-DlK2R^4K^ results in a wildtype phenotype. **B**, **B’** Phenotype of Dl^attP^-Dl-K742R is stronger than that of Dl-heterozygous flies, indicated by the fusion of the tarsal segments 3 + 4. **B’’**, **B’’’** This phenotype is abolished by the addition of a second copy of Dl^attP^-Dl-K742R. Nevertheless, a small amount of extra vein material is still observed, indicating that even two copies cannot restore the wildtype phenotype completely (arrow and arrowhead). For further information, see text

Although more than 6 Ks are required for the full function of Dl, the four core Ks are the most conserved among the Dl orthologs of insect species. Therefore, we wondered whether the presence of only the core Ks in Dl provides sufficient Dl-activity for normal development. We generated *Dl*^*attP*^*-DlK2R*^*4K*^*-HA* and compared its phenotype to that of *Dl*^*attP*^*-Dl-HA*. We found that the phenotype of *Dl*^*attP*^*-DlK2R*^*4K*^ over the deficiency *Dl*^*BSC850*^ was only slightly stronger to that of *Dl*^*attP*^*-Dl-HA *or endogenous *Dl* (Fig. 8A–A’’’, compare with Fig. 3A, A’). However, similar to *Dl*^*attP*^*-Dl-HA*, homozygosity of *Dl*^*attP*^*-DlK2R*^*4K*^ resulted in a wildtype phenotype (Fig. 8A’’, A’’’). Thus, *Dl*^*attP*^*-DlK2R*^*4K*^ is a functional allele, although it has not the full activity in our over-expression analysis. It appears that a window of Dl activity exists that allows correct development. The slightly reduced signalling activity of *Dl*^*attP*^*-DlK2R*^*4K*^ detected in our over-expression experiments is not relevant for development as long as it provides a level of activity that lies in the window.

Our analysis revealed that K742 is the most important K of the ICD. To evaluate its importance for the whole of development of *Drosophila*, we generated the knock-in allele *Dl*^*attP*^*-Dl-K742R-HA*. We found that it is not able to provide the full function of *Dl*, even if present in two copies (Fig. 8B–B’’’). In heterozygosity (*Dl*^*attP*^*-Dl-K742R-HA*/*Df*), the phenotype of *Dl*^*attP*^*-Dl-K742R-HA* was slightly stronger than that of heterozygosity of *Dl* (Fig. 7B, compare with Fig. 3A). Moreover, even in homozygosity, small amounts of extra-vein material were observed (arrow and arrowhead in Fig. 7B’’). This finding confirms the importance of K742, also for the in vivo function of Dl.

## Discussion

Endocytosis of the ligands plays a critical role in the ligand-dependent activation of the Notch pathway. One important way to induce endocytosis is ubi of their ICDs by Mib1 [10, 11, 27]. We have previously shown that human MIB1 binds to JAG1 via a bipartite recognition mode that requires the NB and CB of the ICD of JAG1 and the MZM and REP domains of MIB1 [7]. However, several questions remained unanswered: 1. it was not clear whether this bipartite binding mode is of general importance and also operates in other species, e.g. *Drosophila*, 2. the individual importance of the MZM and REP domains were not investigated, 3. it was not sure that a functional CB exists in Dl, and 4. it was not known, which and how many Ks in the ICD of a DSL ligand are required for its full function. Here, we provided answers to these questions. In addition, we provide further evidence that ubi of Dl by Mib1 is important for signalling and also adjusting/suppressing CI. Moreover, we here show that this ability of Mib1 depends on the binding of its MZM and REP domains to the NB and CB in Dl.

We found that also Dl has a functional NB and CB. So far only the NB was identified [6]. The individual importance of the NB and CB of JAG1 was not determined. We here found that the binding boxes have different importance for function, with the NB being the dominant site. The results suggest that the strength of binding of Mib1 to Dl determines the strength of signalling, since the inactivation of both boxes reduces the activity of Dl. The large dependence of the CB on the presence of the NB and its relatively weak phenotype upon mutation is probably the reason why its importance has not been recognised in a previous study, which used randomly inserted constructs where the position effects are not neutralised [6].

We provide further evidence that, besides the MZM domain, also the REP domain is important for the function of Mib1 in *Drosophila*. Their combined loss resulted in a complete loss of Mib1 function and the ability to activate Dl. Moreover, our results suggest that the CB of Dl interacts with the REP domain in vivo, as it has previously been found for JAG1 in vitro [7]. We also showed that a Dl-variant with mutated NB cannot be activated by Mib1ΔREP, supporting the notion that the NB interacts with the MZM domain of Mib1. A direct interaction of a peptide containing the NB of Dl with the MZM domain of MIB1 has been demonstrated in vitro and our results provide evidence that it occurs in vivo in the context of the full-length proteins, especially as we found that the N in the NB is as important for Mib1-mediated signalling as predicted from the atomic structure. In agreement with these results is also the observation that the MZM domain of Mib1, which interacts with the NB, is more important for function than the REP domain. Our results suggest that the activation of Dl by Mib1 appears to use the same bipartite binding mode originally discovered for interaction of JAG1 with MIB1. Hence, they provide evidence that the bipartite binding mode is the general rule for binding of Mib1 proteins to DSL ligands. Combined with the previous work by McMillan et al. [7] and Daskalaki et al. [6], our findings support a model in which Mib1 contacts the ICD of Dl mainly via its MZM domain binding to the NB and subsequently contacting the CB with its REP domain to consolidate the binding. This bipartite binding results in efficient and selective ubi of Ks in the ICD of Dl. It appears that the importance of the NB and CB of JAG1 for the interaction with MIB1 is more balanced, as it has been shown that the deletion of either the NB or CB results in the abolishment of binding to MIB1 [7].

We identified 6 out of the 12 existing Ks in the ICD of Dl important for Mib1-dependent signalling. Four of these Ks, the core Ks, are highly conserved, with K742 being the most important for the activity of Dl. The individual replacement of the other core Ks by R did not (K688 and K683) or weakly (K755) affect the activity of Dl. Their importance was revealed by the combined replacement/introduction with the other core Ks, especially K742. The previous study by Daskalaki et al. [6] did not identify K742 as important for the Mib1-dependent activity of Dl. In contrast to this study, the previous one used randomly inserted insertions of Dl variants, which was the only available method at that time. The analysis allowed only the detection of large differences in the activity of Dl-variants, because of the varying position effects. Dl^K742R^ still has significant Mib1-dependent activity, which probably obscured the importance of K742 in the previous analysis because of the technical limitations.

One interesting observation is that two core Ks, K688 and K683, are effective upon the re-introduction into DlK2R only in combination with K742 and to a lesser extent with K775. An explanation for this puzzling finding might be that the initial ubi of K742 (and possible K775) is a prerequisite for ubi of the other Ks. One possible scenario would be that the ubi of K742 induces a change in the conformation of the ICD that promotes ubi of the other Ks.

In principle, K742 could also contribute to the binding of Mib1 to the ICD of Dl, as it is located at the N-terminal edge of the CB. However, previous work showed that the deletion of K742 did not have an obvious effect on the ubi of the ICD of Dl, suggesting that Mib1 can still effectively bind to the ICD of Dl in the absence of K742 [6]. Interestingly, previous work that analysed Dl alleles with trafficking defects found that one allele, *Dl*^*CE16*^, had a K742R mutation [32]. However, this allele also bears a mutation in its ECD and the authors showed that also the ECD is somehow involved in endocytosis. Thus, it was not clear which mutation was responsible for the observed trafficking defect. Our work clarifies this issue.

The Ks required for the full activity of Dl in addition to the core Ks, K665 and K762, are less well conserved among the insect orthologs analysed, indicating a certain flexibility in the use of Ks. It appears that already the four core Ks provide the activity of Dl necessary for the Mib1-dependent developmental processes and the additional activity achieved with the ubi of K665 and K762 is not absolutely required, but might provide a certain robustness for signalling. Our finding that *Dl*^*attP*^*-DlK2R*^*4K*^ provided sufficient Dl-activity for normal development supports this notion. It appears there is a threshold of activity required for normal development that can be crossed already with the presence of only the core Ks. Of note here is that we have recently found that already the presence of one copy of the knock-in allele *Dl*^*attP*^*-DlK2R-HA* provides sufficient activity to complete development, giving rise to adult flies with an only slightly enhanced phenotype compared to *Dl* heterozygous flies [16]. This work also revealed that Mib1 activates Dl solely via ubi, whereas Neur can activate Dl also in an ubi-independent manner sufficiently high to allow the correct course of Neur-dependent processes, such as neurogenesis. These findings explain the relatively weak effect of the replacement of all Ks of the ICD of Dl on *Drosophila* development. It also explains why the re-introduction of the core Ks in *Dl*^*attP*^*-DlK2R*^*4K*^*-HA* results in a fully functional allele that completely resembles Dl. In fact, we think that the over-expression of Dl with the Gal4-system exaggerated the effects of loss of Ks and therefore was a more sensitive assay than a knock-in approach to reveal the meaning of the Ks and detect the contribution of the less well conserved Ks to Dl-signalling.

## Conclusions

The work reveals how the ligands of the Notch pathway are activated through the interaction with Mindbomb1, the major E3-ligase devoted to Notch signalling. It reveals that, in *Drosophila*, Mindbomb1 interacts with the ligand Delta in a bipartite mode in vivo, as previously found for the human orthologs JAGGED1 and MINDBOMB1. This finding suggests that the bipartite interaction mode is of general importance for interaction of Mib1 with the Notch ligands. In addition, our work identifies the target sites for the enzymatic activity of Mindbomb1 in the Delta-ligand. Overall, the results contribute to a deeper understanding of how the ligands of the evolutionary Notch-pathway are activated during Notch-signalling in metazoans.

## Methods

### Fly strains

*Dl*^*attP*^*-Dl-NB2A* + *K-HA* FRT82B, *Dl*^*attP*^*-Dl-NB2A-HA* FRT82B, *Dl*^*attP*^*-Dl-CB2A* + *K-HA* FRT82B, *Dl*^*attP*^*-Dl-NB2A-HA* FRT82B, *Dl*^*attP*^ [24], *Dl*^*attP*^*-Dl-HA* FRT82B, *Dl*^*attP*^*-Dl-K2R-HA* FRT82B [16], *mib1*^*EY09870*^ [10, 11], Gbe + Su(H)-lacZ [33], *ptc*GAL4 [34], FRT82B (Bloomington stock centre (BSC), BSC2035), FRT82B ubiRFPnls (BSC30555), FRT82B ubiGFPnls (BSC32655), *Dl*^*rev10*^ e FRT82B [22], Df(3R) BSC850 (BSC27922) [35], *lqf*^*ARI*^ [36], *Dl*^*rev10*^ e *Ser*^*RX82*^ FRT82B [22].

Stocks generated for this study: *Dl*^*attP*^*-Dl-NB2A-HA* FRT82B, *Dl*^*attP*^*-Dl-NB2A-HA* FRT82B, *Dl*^*attP*^*-Dl-CB2A-HA* FRT82B, *Dl*^*attP*^*-Dl-NB2A-HA* FRT82B, *Dl*^*attP*^*-Dl-NB* + *CB2A-HA FRT82B*, *Dl*^*attP*^*-Dl-NB* + *CB2A-HA* FRT82B, variants where K were re-introduced into *Dl*^*attP*^*-DlK2R-HA* FRT82B and variants where Ks were replaced by Rs in *Dl*^*attP*^*-Dl-HA* FRT82B, *Dl*^*attP*^*-Dl-NB2A-HA* FRT82B, *Dl*^*attP*^*-Dl-CB2A-HA* FRT82B, *Dl*^*attP*^*-Dl-NB* + *CB2A-HA* FRT82B, *Dl*^*attP*^*-Dl-ΔICD-HA* FRT82B *Dl*^*attP*^*-HA-Dl* FRT82B, *Dl*^*attP*^*-Dl-DlK2R*^*4K*^*-HA* FRT82B, *Dl*^*attP*^*-Dl-K742R-HA* FRT82B. *tubP-mib1ΔMZM* + *REP*, *tubP-mib1REP*, *tubP-mib1*.

### Quantification of ectopic Wg activation and cis-inhibition (CI)

Quantification was performed using the software ImageJ. The length of CI was measured and divided by the length of endogenous Wg expression (CI/endogenous Wg). The result indicates the percentage of CI that is acting on the endogenous Wg.

Ectopic activation of Wg was measured by assessing the length of the anterior and/or posterior Wg expression and addition of the lengths. The added lengths were divided against the endogenous Wg. The result indicates the fold-change of ectopic Wg expression relative to the endogenous Wg expression. The significance was determined by using an unpaired, two-samples *t*-test with H_0_ = the means of the two samples do not differ; with the following significance-levels: *p* > 0.05 = ns; *p* < 0.05 = *; *p* < 0.01 = **; *p* < 0.001 = ***.

### Antibody staining and imaging

Antibody staining was performed according to standard protocols [37] and [38].

### Antibodies used

**Table:** 

Antibody list		
Antibody (from species)	Source or reference	Dilution
Anti HA (rat)	Roche Clone 3F10	1:500
Anti HA (rabbit)	Cell Signaling C29F4	1:1600
Anti Dl (mouse)	DSHB C594.9B	1:500
Anti Dl (mouse)	DSHB C594.9B	1:100 (surface staining)
Anti N(extra) (mouse)	DSHB C458.2H	1:100
Anti Wg (mouse)	DSHB 4D4	1:250
Anti ß-Gal (rabbit)	Cappel	1:1500
Anti ß-Gal (rabbit)	Cell Signaling A257	1:1500
Anti ß-Gal (mouse)	DSHB 40-1a	1:250
Anti Rab11 (rabbit)	Tanaka and Nakamura 2008	1:8000
Anti Hnt (mouse)	DSHB 1G9	1:80
Anti Rabbit Alexa 488 (goat)	Invitrogen/Molecular Probes	1:500
Anti Rabbit Alexa 568 (goat)	Invitrogen/Molecular Probes	1:500
Anti Rabbit Alexa 647 (goat)	Invitrogen/Molecular Probes	1:500
Anti Mouse Alexa 488 (goat)	Invitrogen/Molecular Probes	1:500
Anti Mouse Alexa 568 (goat)	Invitrogen/Molecular Probes	1:500
Anti Mouse Alexa 647 (goat)	Invitrogen/Molecular Probes	1:500
Anti Rat Alexa 488 (goat)	Invitrogen/Molecular Probes	1:500
Anti Rat Alexa 568 (goat)	Invitrogen/Molecular Probes	1:500
Anti Rat Alexa 647 (goat)	Invitrogen/Molecular Probes	1:500

### Generation of constructs

#### Generation of UAS Dl-variants

Dl-HA was generated by introduction of a synthesised fragment from the NdeI restriction site of the ICD onwards. The DlK2R-HA was generated by replacing the ICD of Dl-HA by a synthesised ICD in which all Ks are replaced by Rs. Gene synthesis was performed by GenScript. Single point mutations of each K were introduced by site directed mutagenesis (SDM) with the Pfu Polymerase by Promega and complementary primers. Each K was replaced by an R. All constructs were cloned into pUAST attB by BstEII and NdeI.

Mib1-binding mutants (NB2A, CB2A and NB + CB2A) were also generated via SDM. The Dl-constructs were injected into the landing site 51C. Transgenesis was partly performed by BestGene Inc.

#### Generation of tubP-(V5-)Mib1 variants

pBluescript (pBS) *mib1* was generated by introduction of the amplified cDNA of *mib1* (gift from C. Delidakis) into pBluescript by EcoRI and XhoI. *mib1* was amplified with an N-terminal primer inserting a NotI restriction site and the V5 tag and cloned into pattB tub by NotI and XhoI. MZM or REP domains were deleted by site directed mutagenesis in pBS *mib1*. The mutated areas of Mib1 variants were cloned into pattB tub V5 mib1 by BbvCI and StuI. The tub.Mib1 variants were inserted into the 22A landing site.

Generation of the D|^attP^-variants: D|^attP^-variants were generated by cloning the corresponding variant from the pUAST attB-vector into the pGE-vector that was generated in our previous study by employing the restriction sites BstEll and Xbal [16]. This replaces the trans-membrane domain and the whole ICD. The vectors were injected into *Dl*^*attP*^ [24]. *D|*^*attP*^*-ΔICD-HA* was synthesised by IDT technology, bearing 12aa from the N-terminal ICD to ensure proper incorporation into the plasma membrane. Ks within these 12aa were replaced by Rs.

Alleles generated: *D|*^*attP*^*-NB2A-HA*, *D|*^*attP*^*-CB2A-HA*, *D|*^*attP*^*-NB* + *CB2A-HA*, *D|*^*attP*^*-Dl-K2R*^*4K*^ (K683, K688, K742, K775), *D|*^*attP*^*-K742R-HA*, *D|*^*attP*^*-ΔICD-HA*. All constructs were sequenced before injection into *D|*^*attP*^.

The following primers were used to generate the Dl- and Mib1-constructs:

Dl-variants

**Table:** 

**Mutation**	**Name**	**Sequence (5′** à **3′)**
K620, 622, 624R	Dl_sdm_KTMR_fwd	C TTC TGC ATG AGG CGC AGG CGT AGG CGT GCT CAG
	Dl_sdm_KTMR_rev	CTG AGC ACG CCT ACG CCT GCG CCT CAT GCA GAA G
K629R	Dl_K629R_Fwd	CGT GCT CAG GAA AGG GAC GAC GCG GAG
	Dl_K629R_Rev	CTC CGC GTC GTC CCT TTC CTG AGC ACG
K636R	Dl_K636R_Fwd	GAC GCG GAG GCC AGG AGG CAG AAC GAA CAG
	Dl_K636R_Rev	CTG TTC GTT CTG CCT CCT GGC CTC CGC GTC
K665R	Dl_K665R_Fwd	C TCT ATG GGC GGC AGA ACT GGC AGC AAC AG
	Dl_K665R_Rev	CT GTT GCT GCC AGT TCT GCC GCC CAT AGA G
K739R	Dl_K739R_Fwd	GT GTG GCT CCG CTA CAA AGA GCC AGG TCG C
	Dl_K739R_Rev	G CGA CCT GGC TCT TTG TAG CGG AGC CAC AC
K762R	Dl_K762R_Fwd	CA GGC AGC TCA GCC AGG GGA GCG TCT GGC G
	Dl_K762R_Rev	C GCC AGA CGC TCC CCT GGC TGA GCT GCC TG
K775R	Dl_K775R_Fwd	CG GCG GAG GGC AGG AGG ATC TCT GTT TTA G
	Dl_K775R_Rev	C TAA AAC AGA GAT CCT CCT GCC CTC CGC CG

K2R-variants

**Table:** 

R620, 622, 624 K	DlK2R_sdm_RTMK_fwd	C TTC TGC ATG AAA CGC AAG CGT AAG CGT GCT CAG
	DlK2R_sdm_RTMK_rev	CTG AGC ACG CTT ACG CTT GCG TTT CAT GCA GAA G
R629K	DlK2R_R629K_Fwd	CGT GCT CAG GAA AAA GAC GAC GCG GAG GCC
	DlK2R_R629K_Rev	GGC CTC CGC GTC GTC TTT TTC CTG AGC ACG
R636K	DlK2R_R636K_for	GAC GAC GCG GAG GCC AGG AAG CAG AAC GAA CAG
	DlK2R_R636K_rev	CTG TTC GTT CTG CTT CCT GGC CTC CGC GTC GTC
R665K	DlK2R_R665K_for	C TCT CTG GGC GGC AAG ACT GGC AGC AAC AG
	DlK2R_R665K_rev	CT GTT GCT GCC AGT CTT GCC GCC CAG AGA G
R739K	DlK2R_R739K_for	GT GTG GCT CCG CTA CAA AGA GCC AAG TCG C
	DlK2R_R739K_rev	G CGA CTT GGC TCT TTG TAG CGG AGC CAC AC
R762K	DlK2R_R762K_for	CA GGC AGC TCA GCC AAA GGA GCG TCT GGC G
	DlK2R_R762K_rev	C GCC AGA CGC TCC TTT GGC TGA GCT GCC TG
R775K	DlK2R_K775R_Fwd	CG GCG GAG GGC AAG AGG ATC TCT GTT TTA GG
	DlK2R_K775R_Rev	CC TAA AAC AGA GAT CCT CTT GCC CTC CGC CG

Boxes Dl

**Table:** 

Mutation	Name	Sequence (5′ à 3′)
Deletion of ICD (AS 618–833)	Dl_sdm_ΔICD_for	G TGC GTG GTC TTC GCC TAC CCA TAC
	Dl_sdm_ΔICD_rev	GTA TGG GTA GGC GAA GAC CAC GCA C
N684A	Dl_sdm_N684A_fwd	C CCG AAT ATC ATC AAA GCC ACC TGG GAC AAG TCG GTC
	Dl_sdm_N684A_rev	GAC CGA CTT GTC CCA GGT GGC TTT GAT GAT ATT CGG G
IKNTWDK to AAAAAAA	Dl_sdm_NBox2A_fwd	GGC GGC AAC CCG AAT ATC GCC GCA GCC GCC GCG GCC GCG TCG GTC AAC AAC ATT TGT GCC
	Dl_sdm_NBox2A_rev	GGC ACA AAT GTT GTT GAC CGA CGC GGC CGC GGC GGC TGC GGC GAT ATT CGG GTT GCC GCC
IKNTWDK to AKAAAAK	Dl_sdm_NBox2A_K_fwd	GC GGC AAC CCG AAT ATC GCC AAA GCC GCC GCG GCC AAG TCG GTC AAC AAC
	Dl_sdm_NBox2A_K_rev	GTT GTT GAC CGA CTT GGC CGC GGC GGC TTT GGC GAT ATT CGG GTT GCC GC
IKNTWDK to AANAAAA	Dl_sdm_N-Box2A-N684_for	GGC GGC AAC CCG AAT ATC GCC GCA AAC GCC GCG GCC GCG TCG GTC AAC AAC ATT TGT G
	Dl_sdm_N-Box2A-N684_rev	C ACA AAT GTT GTT GAC CGA CGC GGC CGC GGC GTT TGC GGC GAT ATT CGG GTT GCC GCC
KQLNTD to AAAAAA	Dl_sdm_CBox2A_fwd	CAA AGA GCC AAG TCG CAA GCC GCA GCC GCC GCC GCT CCC ACG CTC ATG CAC CG
	Dl_sdm_CBox2A_rev	CG GTG CAT GAG CGT GGG AGC GGC GGC GGC TGC GGC TTG CGA CTT GGC TCT TTG
KQLNTD to KAAAAA	Dl_sdm_CBox2A_K_fwd	GCC AAG TCG CAA AAG GCA GCC GCC GCC GCT CCC ACG CTC ATG CAC C
	Dl_sdm_CBox2A_K_rev	G GTG CAT GAG CGT GGG AGC GGC GGC GGC TGC CTT TTG CGA CTT GGC
IKNTWDKSVNNI to IKNTWAKSVAAA	Dl_sdm_DNNI2A_for	C AAA AAC ACC TGG GCC AAG TCG GTC GCC GCC GCT TGT GCC TCA GCA G
	Dl_sdm_DNNI2A_rev	C TGC TGA GGC ACA AGC GGC GGC GAC CGA CTT GGC CCA GGT GTT TTT G

Mib1

**Table:** 

Name	Sequence (5′ à 3′)
mib1_NotI_V5_forward	G TGA CCA GGC GGC CGC ATG ATC CCT AAC CCT CTC CTC TCT TGT GCG GCC ACC C NotI-restriction site, start-codon, V5-tag, binds mib1
XhoI-Dmib1_backward (E. Seib)	CAC TAG CTC GAG TCA GAA GAG CAG GAT GCG XhoI-restriction site, binds mib1

**Table:** 

Mutation	Name	Sequence (5′ à 3′)
Deletion of the MZM-domain (AS 100–315)	dMib1_sdm_dMZM_for	CG GCA GCG GCC CTC AAG GGT AGT AAT GTT TAC
	dMib1_sdm_dMZM_rev	GTA AAC ATT ACT ACC CTT GAG GGC CGC TGC CG
Deletion of the REP-domain (AS 333–487)	dMib1_sdm_dREP_for	CTG GGC GAA AAC GGA TCA ACT GCC TCC G
	dMib1_sdm_dREP_rev	C GGA GGC AGT TGA TCC GTT TTC GCC CAG

Primers were purchased from Sigma-Aldrich.

## Supplementary Information

Additional file 1: Figures S1–S7. Fig. S1 Comparison of the aa sequence of the ICD of Dl orthologs among insect species. Fig. S2 The phenotype of expression of Dl-NB2A and Dl-CB2A with the flanking Ks also exchanged to A. Fig. S3 The phenotype of a Dl knock-in allele with the HA tag inserted into the extracellular domain, close to the transmembrane domain. Fig. S4 The role of the ICD of Dl revealed by the analysis of DlattP-DlΔICD-HA. Fig. S5 The adult phenotype of mib1 mutant flies rescued with one copy of the described Mib1 variants expressed under control of tub.P. Fig. S6 Presentation of the complete analysis of all Dl variants generated and tested for this study. Fig. S7 The role of the Ks in the ICD of Dl close to the transmembrane domain.

## Data Availability

No datasets were generated or analysed during the current study.

## Abbreviations

Dl: Delta
Mib1: Mindbomb1
NB: N-box
CB: C-box
MZM: Mib-Herc2-ZZ **z**inkfinger-Mib1-Herc2 domain
REP: Mib repeat-Mib repeat domain
Neur: Neuralized
Ks: Lysines
JAG1: JAGGED1
ubi: Ubiquitylation
*kuz*: *Kuzbanian*
NEXT: Notch EXtracellular Truncation
NICD: Notch intracellular domain
Su(H): Suppressor of Hairless
ICDs: Intracellular domains
*wg*: *Wingless*
R: Arginine
Dll1: Delta-like1
*ptc*Gal4: *patched*-Gal4
A/P-boundary: Anterior-posterior compartment boundary
D/V-boundary: Dorso-ventral compartment boundary
N: Asparagine
