# Supplementary material for: Another tail of two sites: activation of the Notch ligand Delta by Mindbomb1
Source: BMC Biol. 2025 Mar 6;23:71. doi: 10.1186/s12915-025-02162-6 (PMC11887331; doi:10.1186/s12915-025-02162-6)
Supplement: Supplementary file 1 — Additional file 1: Figures S1–S7. Fig. S1 Comparison of the aa sequence of the ICD of Dl orthologs among insect species. Fig. S2 The phenotype of expression of Dl-NB2A and Dl-CB2A with the flanking Ks also exchanged to A. Fig. S3 The phenotype of a Dl knock-in allele with the HA tag inserted into the extracellular domain, close to the transmembrane domain. Fig. S4 The role of the ICD of Dl revealed by the analysis of DlattP-DlΔICD-HA. Fig. S5 The adult phenotype of mib1 mutant flies rescued with one copy of the described Mib1 variants expressed under control of tub.P. Fig. S6 Presentation of the complete analysis of all Dl variants generated and tested for this study. Fig. S7 The role of the Ks in the ICD of Dl close to the transmembrane domain. [file 12915_2025_2162_MOESM1_ESM.docx]

**Additional file 1: Dataset supplemental figures.**

***Fig. S1****. (A) Comparison of the aa sequence of the ICD of Dl orthologs among insect species. The arrows highlight the Ks in the ICD of Dl. The green arrows highlight the most conserved Ks. (B) Cartoon of the ICD of Dl revealing the location of the NB and CB and the Ks. Note that K683 and K688 flank the NB and K742 is located at the N-terminus of the CB.*

*
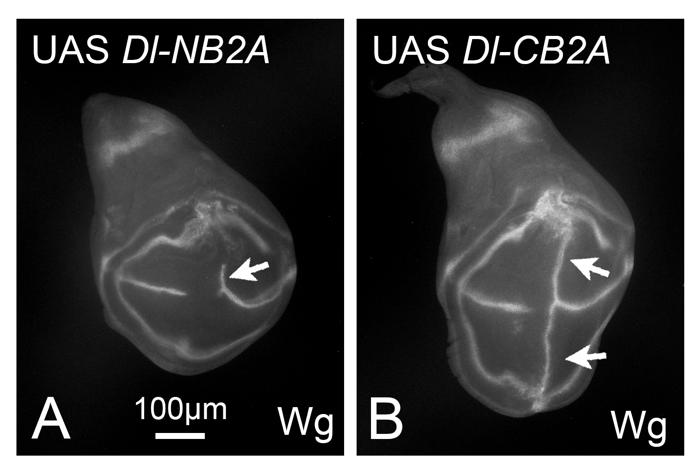
*

***Fig. S2****. The phenotype of expression of Dl-NB2A and Dl-CB2A with the flanking Ks also exchanged to A. Compare with the variants that posses the Ks, shown in Fig. 2D and E. No significant difference in the phenotype is observed.*

*
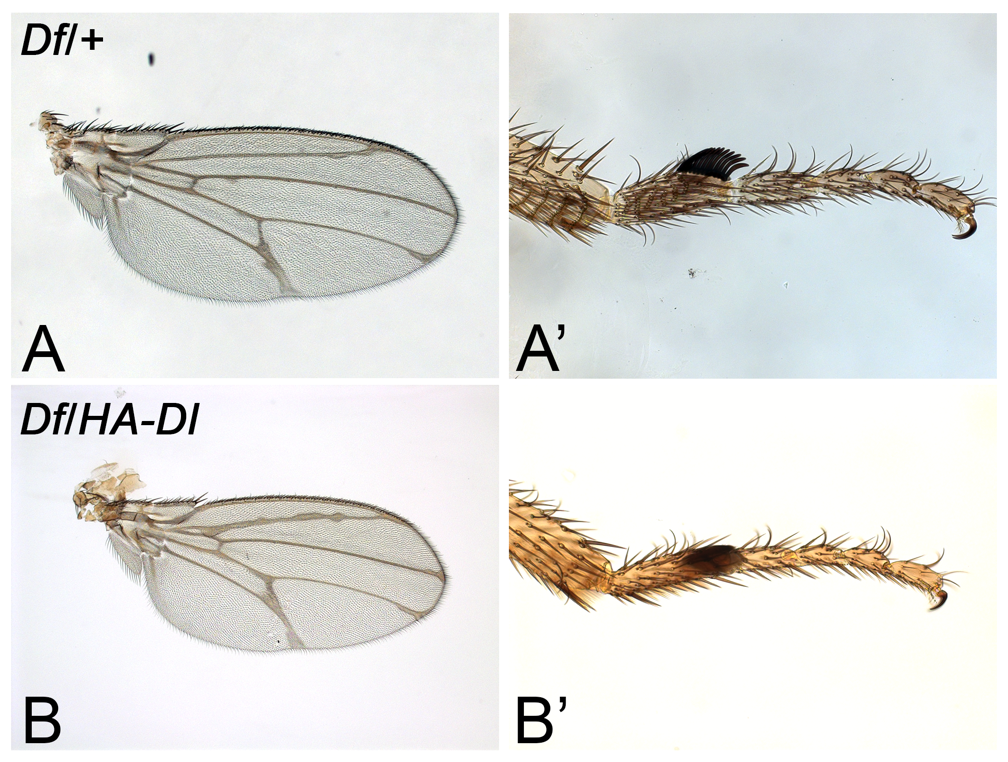
*

***Fig. S3****. (A) The phenotype of a Dl knock-in allele with the HA tag inserted into the extracellular domain (Dl^attP^-HA-Dl), close to the transmembrane domain. (A, A’) the Wing (A) and leg (A’) of a fly carrying the Dl-deficiency Dl^BSC850^ in heterozygousity, displaying the dominant Dl wing phenotype. The leg is wildtype in appearance. (B, B’) Phenotype of Dl^attP^-HA-Dl over the deficiency displays a comparable wing (B) and leg (B’) phenotype. Compare also to the phenotype of Dl^attP^-Dl-HA, which encodes the variant with a HA tag at the C-terminus displayed in Fig. 3A. The results indicate that the addition of the tag to the C-terminus does not affect the activity of Dl.*

*
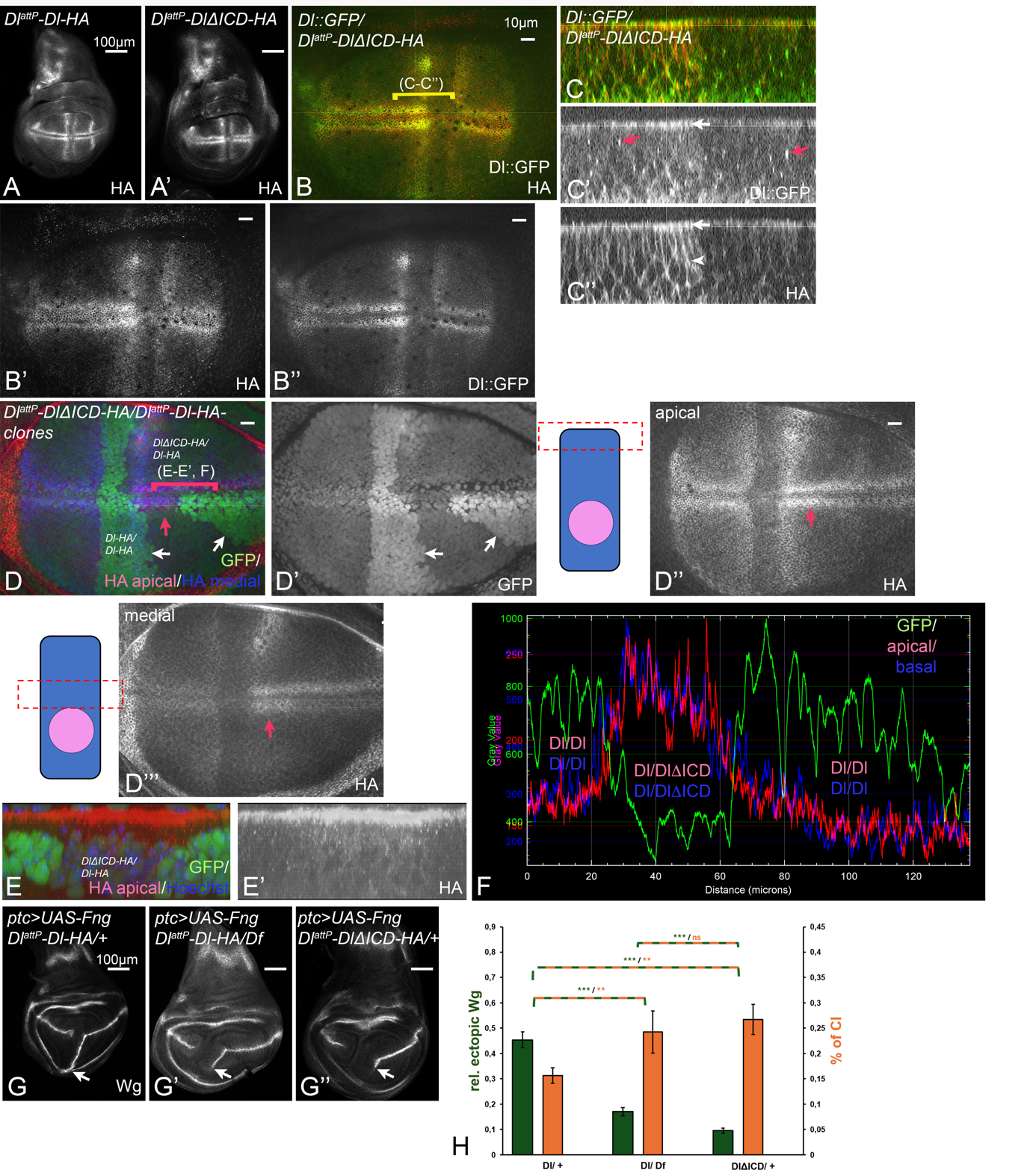
*

***Fig. S4.*** *The role of the ICD of Dl revealed by the analysis of DlattP-DlΔICD-HA.* *(A-C’’) Expression of DlΔICD-HA in comparison to Dl::GFP. (A, A’) DlΔICD-HA is expressed in the same pattern as Dl::GFP, indicating that it is expressed correctly. (B-B’’) enlargement of the wing area of the discs shown in (A, A’) The focus is on the apical region of the plasma membrane. It indicates that DlΔICD-is localised in the same region as Dl::GFP. (C-C’’) Z-section of the region highlighted with the yellow bracket in (B). It confirms the localisation of DlΔICD-HA in the same apical domain of the plasma membrane as Dl::GFP (white arrow in C’, C’’). In contrast to Dl::GFP, DlΔICD-HA is also localised in the basolateral membrane domain (arrowhead in (C’’) and virtually absent from the Dl:GFP-positive intracellular punctae highlighted by the red arrows in (C’). The analysis indicates that DlΔICD-HA is efficiently exocytosed, but fails to undergo endocytosis. Hence the ICD is required only for endocytosis of Dl. (D-F) Clonal analysis of Dl^attP^-DlΔICD-HA. Homozygous Dl^attP^-DlΔICD-HA and Dl^attP^-Dl-HA twin clones were induced as described in Fig. 4A. (D-D’’’) Wing area of a disc where clones are induced. The Dl-HA homozygous clones are labelled by two copies of GFP, the Dl-HA/ DlΔICD-HA heterozygous cells with one copy of GFP and the DlΔICD-HA homozygous clones by absence of GFP. The disc contains only heterozygous and Dl-HA homozygous clones (2x GFP, arrows). (D’) No DlΔICD-HA homozygous clones can be detected, indicating that DlΔICD-HA is cell lethal in homozygousity. (D’’, D’’’) Comparison of the expression of DlΔICD-HA/Dl-HA compared to homozygous Dl-HA clones. (D’’) Focus on the apical membrane domain as highlighted by the cartoon. (D’’’) Focus on the lateral membrane domain as highlighted by the cartoon. (F) Pixel density plot of the region highlighted in (D) by the red bracket. It reveals that the HA signal in the Dl-HA homozygous clones (2xGFP) is weaker in the Dl homozygous cells. This indicates that the DlΔICD-HA, present in the heterozygous cells accumulates to a higher level than Dl-HA. (G-G’’) Ectopic expression of Fng with ptcGal4 in wing discs of Dl-HA/+ (G), Dl-HA/Df (G’, heterozygous for Dl) and DlΔICD-HA/+ (G’’) genotype. (H) Quantification of the length of the induced ectopic stripe highlighted by the arrow in (G-G’’). The analysis shows that the ectopic stripe of Wg induced in the DlΔICD-HA heterozygous disc (G’’) is significantly shorter than that induced in Dl heterozygous discs (G’), indicating that DlΔICD-HA has a negative effect on the induction of ectopic Wg expression by Dl. Combined the results indicate that DlΔICD-HA acts in a dominant-negative manner.*

*´*
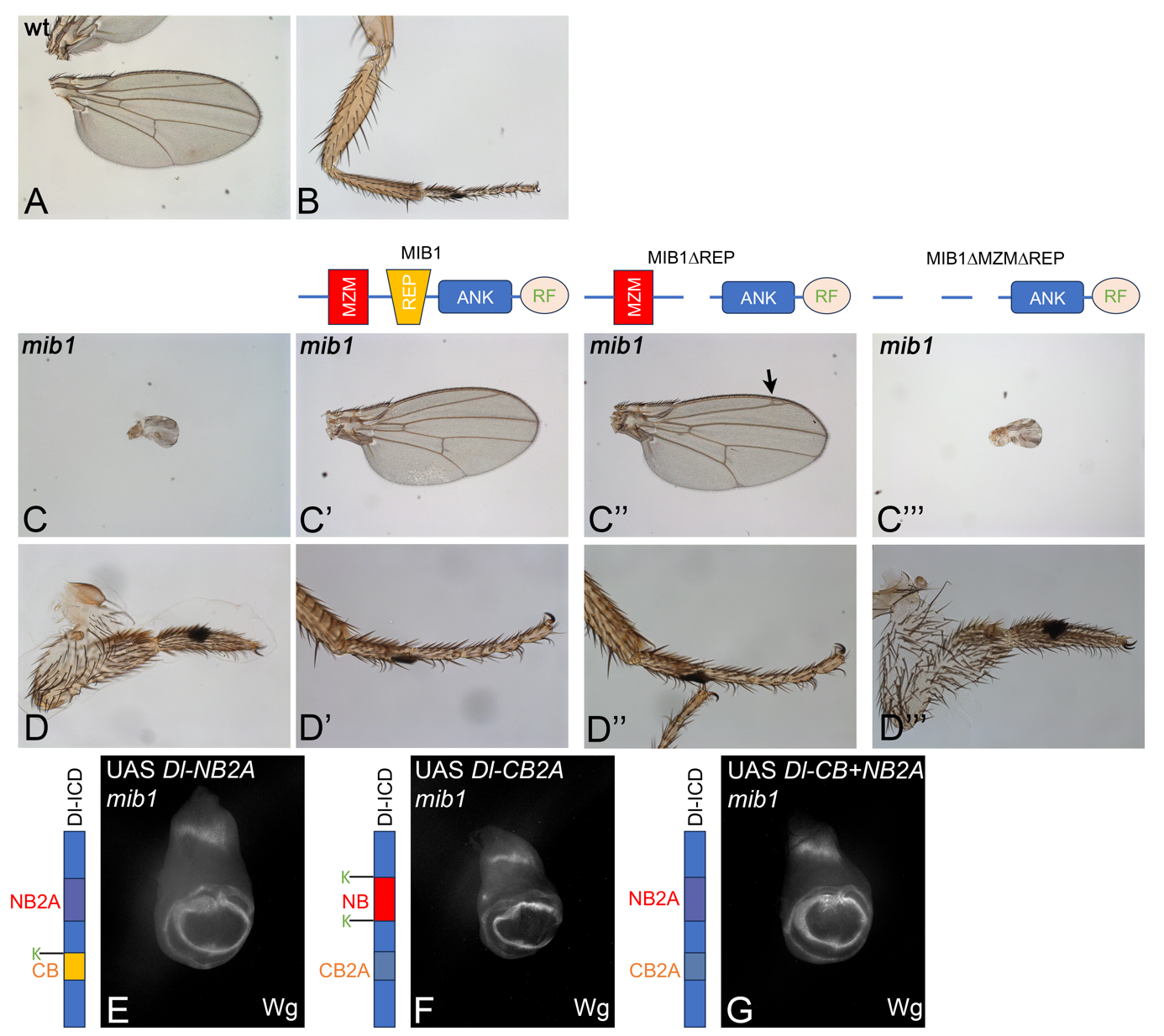


***Fig. S5****. (A-D’’’) The adult phenotype of mib1 mutant flies rescued with one copy of the described Mib1 variants expressed under control of tub.P. Note, that Mib1ΔREP rescues nearly as good as Mib1, while Mib1ΔMZMΔREP fails to rescue and resembles the mib1 mutant phenotype. (E-G) Expression of Dl-variants in mib1 mutants fail to significantly induce Wg expression.*


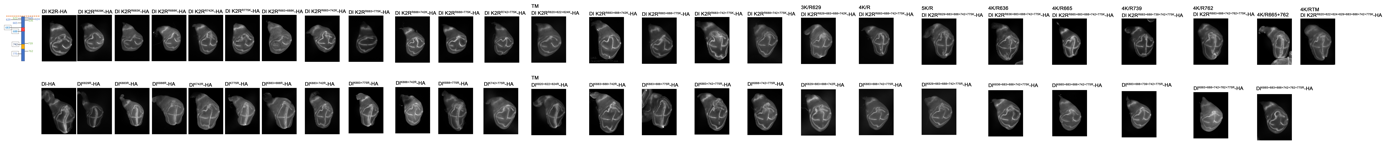


***Fig. S6****. Presentation of the complete analysis of all Dl variants generated and tested for this study. For further information, see text.*

*
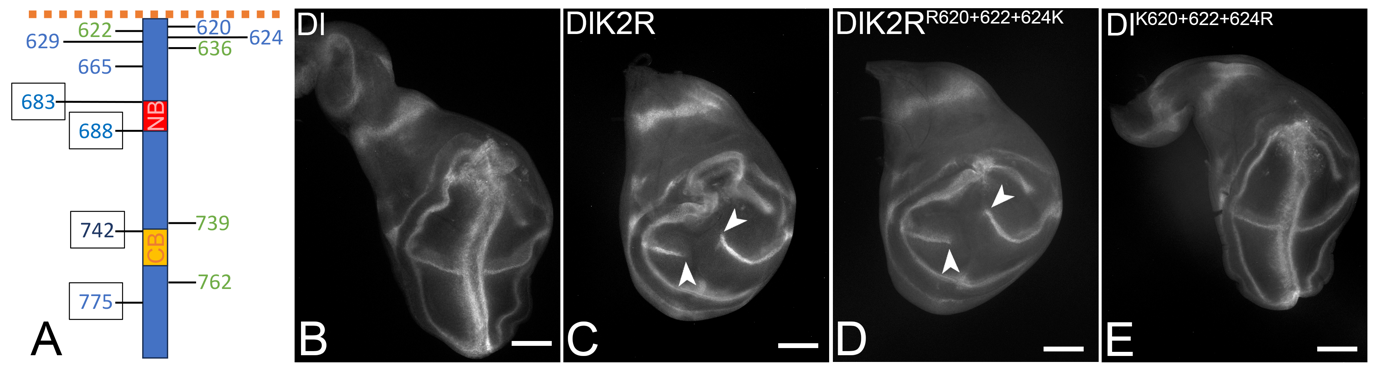
*

***Fig. S7****. The role of the Ks in the ICD of Dl close to the transmembrane domain. (A) Cartoon of the location of the Ks in the ICD of Dl. (B-E) Neither the re-introduction of the three Ks into DlK2R, nor the exchange to R in Dl change the activity of DlK2R or Dl, respectively (compare with Fig. 1D, F) . For further information, see text.*
